# Recipient-independent, high-accuracy FMT-response prediction and optimization in mice and humans

**DOI:** 10.1186/s40168-023-01623-w

**Published:** 2023-08-14

**Authors:** Oshrit Shtossel, Sondra Turjeman, Alona Riumin, Michael R. Goldberg, Arnon Elizur, Yarin Bekor, Hadar Mor, Omry Koren, Yoram Louzoun

**Affiliations:** 1 Department of Mathematics, Bar-Ilan University, Ramat Gan, 52900 Israel; 2 The Azrieli Faculty of Medicine, Bar-Ilan University, Safed, Israel; 3 Yitzhak Shamir Medical Center (Assaf Harofeh), Zerifin, Israel; 4 Department of Pediatrics, Sackler Faculty of Medicine, Tel Aviv, Israel

## Abstract

**Background:**

Some microbiota compositions are associated with negative outcomes, including among others, obesity, allergies, and the failure to respond to treatment. Microbiota manipulation or supplementation can restore a community associated with a healthy condition. Such interventions are typically probiotics or fecal microbiota transplantation (FMT). FMT donor selection is currently based on donor phenotype, rather than the anticipated microbiota composition in the recipient and associated health benefits. However, the donor and post-transplant recipient conditions differ drastically. We here propose an algorithm to identify ideal donors and predict the expected outcome of FMT based on donor microbiome alone. We also demonstrate how to optimize FMT for different required outcomes.

**Results:**

We show, using multiple microbiome properties, that donor and post-transplant recipient microbiota differ widely and propose a tool to predict the recipient post-transplant condition (engraftment success and clinical outcome), using only the donors’ microbiome and, when available, demographics for transplantations from humans to either mice or other humans (with or without antibiotic pre-treatment). We validated the predictor using a de novo FMT experiment highlighting the possibility of choosing transplants that optimize an array of required goals.

We then extend the method to characterize a best-planned transplant (bacterial cocktail) by combining the predictor and a generative genetic algorithm (GA). We further show that a limited number of taxa is enough for an FMT to produce a desired microbiome or phenotype.

**Conclusions:**

Off-the-shelf FMT requires recipient-independent optimized FMT selection. Such a transplant can be from an optimal donor or from a cultured set of microbes. We have here shown the feasibility of both types of manipulations in mouse and human recipients.

Video Abstract

**Supplementary Information:**

The online version contains supplementary material available at 10.1186/s40168-023-01623-w.

## Background

In recent decades, the relationship between the human gut microbiota and its host health has been shown in a wide range of conditions, ranging from metabolic disorders through autoimmune diseases to mental health disorders [1–6]. The relationship is not only correlative: fecal microbiota transplantation (FMT) has been successful in transferring phenotypes to germ-free (GF) mice in a range of experiments [7–16]. Several studies have found connections between disease states, specific bacterial taxa, and key metabolic pathways directly associated with these taxa; however, the transition to specific mechanisms of action to treat or completely prevent ailments remains elusive.

Often, finding specific bacterial strains or metabolites to prescribe is challenging, as the microbiota is a complex community. Furthermore, clinical studies include real-life noise, unlike experimental setups with GF animals or under extremely controlled environments. Ensuring subsequent colonization of a single strain in the milieu of other microbes is challenging, and predicting the specific microbe’s functionality within the complex microbiota is nearly impossible. Full personalization of microbe-based treatments is still cost prohibitive and may even be unnecessary.

Restorative FMT is a common treatment for CDI (*Clostridioides difficile* infection) and is studied in a large variety of other conditions [17]. FMT is the transfer of a donor’s gut microbiome content to a recipient. In human recipients, transplantation is often performed after a course of antibiotics to clear out the recipient’s own dysbiotic microbiome, thus increasing the probability of colonization [18, 19]. In mice, FMT is most often performed on GF or antibiotic-treated mice, though studies in untreated animals are growing.

Early evidence of FMT dates back nearly 2000 years [20] with more recent anecdotal use of the method, during World War II, among soldiers, to prevent diarrhea [21]. Formally, the use of FMT in the medical field was first documented in 1958 [22]. With the advent of microbiome research, this treatment method has regained clinical interest [23, 24]. Currently, FMT has been clinically trialed and approved to treat recurring CDI [25]. Ongoing clinical research includes the following: improving the response to immunotherapy treatment [26], improving the quality-of-life of autistic children [27], maintaining weight loss [16], and even restoring the normal neonate microbiome after birth via cesarean section [28].

A successful FMT is often characterized by an improvement in some quantitative disease symptom(s) or pathology, but other technical markers, like colonization success (overall richness or of specific microbes), can also be used to mark success or predict efficacy [29]. In general, a healthy microbiome is a diverse one [30–33]; thus, an ideal donor may be one that induces a high microbial richness in the recipient, and a successful FMT would be one in which a maximum number of key microbial taxa colonize the recipient’s gut [34], although recent studies have also demonstrated that the abundance of certain species might be just as important as overall richness [35, 36]. Finally, one may aim to choose an FMT donor not based on post-transplant microbiome but rather associated with a post-transplant condition (fewer clinical symptoms) [37].

Tools to help identify key donors that will likely provide rich microbiota colonization are of high relevance in clinical practice, and the ability to predict a priori which taxa are most likely to engraft from complex and diverse donor microbiota can be helpful in selecting donors for diseases in which “beneficial” microbes have already been identified. However, evidence suggests that colonization of the donor microbiota in the host is not always linear [26], and the mechanisms and dynamics dictating which donor microbes can be engrafted in the recipient are poorly understood.

Initial studies that are able to track the transmission of donor strains to the recipient have been performed on only very few donor-recipient pairs [38]. The completion of larger FMT trials and advances in strain-level metagenomics will enable deeper analyses to unravel general FMT engraftment efficiency patterns across diseases and may lead to the development of statistical models to predict the post-FMT microbiome composition [36], but in the absence of such rich datasets, a more nuanced approach than linear assumptions based on donor richness and abundance profiles is required.

Current approaches in modeling engraftment have two main limitations: first, the vast majority of previous investigations remained confined to single cohorts [36, 39–43], with limited cross-cohort and cross-condition generalizability.

Second, even existing cross-cohort manipulation outcomes require information on the recipient. For example, a recent systematic meta-analysis of 24 studies that investigated FMT in different clinical settings and for which some post-FMT recipient outcomes, such as Shannon diversity, species composition, and species presence were provided, predicted these outcomes using both the donor and recipient (baseline) microbiome and demographic data [35]. Such methods cannot be used for off-the-shelf solutions.

Off-the-shelf solutions or one-size-fits-all treatments (at least for a certain family of diseases), which is one of the major ambitions in the clinic [44], require recipient-independent optimization of the FMT. We here test whether such an optimization is possible, using only donor data to predict species richness and taxa prevalence and abundance in FMT-recipient mice and humans. We then extend the analysis to the prediction of transplant clinical outcomes beyond microbiome properties in humans. Finally, we use this approach to reconstruct an ideal synthetic microbiota that could theoretically be used for microbiota manipulation instead of fecal matter from a human donor.

To that end, we developed iMic (image microbiome) an algorithm to predict transplant outcomes (either engraftment success or the improvement in clinical conditions), based on microbial characterization of the human fecal donor samples alone. iMic is developed for mouse and human recipients. We then validated our model by performing an FMT experiment in antibiotic-treated mice, transplanting ideal and sub-optimal human donor samples, as identified by iMic. These models were then combined and extended in a genetic algorithm (GA) to predict optimal synthetic off-the-shelf microbiome compositions for transplants.

## Methods

### Experimental datasets

We built algorithms based on microbiome data (16S rRNA sequences) from human-to-GF transplants from 1 unpublished and 3 published FMT experiments, where human fecal matter was transplanted to GF mice, and in human-to-human transplants (see Tables 1 and 2). The experiments are described in more detail in the Supplementary Methods, but the human-to-GF cohorts included FMT of stools from patients with gestational diabetes [12], food allergy (unpublished data), antibiotic exposure [13], and undergoing chemotherapy [15]. Six more human-to-human cohorts with clear clinical outcomes of improving a variety of clinical symptoms (e.g., inflammatory bowel disease (IBD) [37], as measured by Mayo score [45], the response to PD-1 therapy in patients with melanoma and others) were analyzed (2 16S cohorts and 4 shotgun metagenomics, see Table 3). Stool samples from mice were collected weekly following FMT and characterized by sequencing the V4 region of 16S rRNA gene, as described in the Supplementary Methods. Some of the published datasets were downloaded from the NCBI (National Center for Biotechnology Information) website via our homemade microbiome downloading and analysis package named YAMAS https://github.com/YarinBekor/YaMAS, also available through PyPI https://pypi.org/project/YMS/.

**Table 1:** Characteristics of all human-to-GF cohorts

Name	Condition	Number of FMTs	Reference
GDM	GDM	30	[12]
Allergy	IgE-mediated food allergies	18	[46]
Chemotherapy	Chemotherapy for breast cancer	159	[15]
Baby	Antibiotic treatment during the neonatal period	48	[13]

**Table 2:** Characteristics of all published human-to-human datasets

Accession number	Disease	Abx	Sample size	Number of FMTs	16S region	Reference
ERP021216	CDI	T	86	20	V4	[47]
PRJDB4959	IBD	F	28	10	V1V2	[48]
PRJNA221789	CDI	T	20	10	V1-V3	[49]
PRJNA238042	CDI	T	22	11	V3-V5	[50]
PRJNA238486	CDI	T	23	3	V6	[51]
PRJNA380944	IBD	T	83	21	V4	[52]
PRJNA412501	IBD	T	52	19	V3V4	[53]
PRJNA428898	IBD	F	35	9	V4V5	[54]

**Table 3:** Characteristics of all published human-to-human cohorts with a clear clinical outcome

Accesstion number	Disease	Success definition	Number of FMTs	16S vs WGS	Reference
PRJEB46777	UC	Simple clinical colitis activity index scores ($\le 2$)	43	WGS	[55]
PRJEB46779	Antibiotics resistance (AR)	Decline in the number of symptoms	33	WGS	[55]
PRJNA672867	Melanoma	Response to PD-1 therapy	26	WGS	[56]
PRJEB36140	IBS	Decline in the number of symptoms	30	WGS	[57]
CRA004875	IBD	Mayo score	103	16S	[37]

### ML nomenclature

In order to facilitate the understanding of the more machine learning (ML)-oriented terms in the text, we here provide a short description of the main ML terms used in the manuscript.

- *Model*. The model is the mathematical relation between any input (in our case the donor microbiome amplicon sequence variants―ASVs) and the appropriate output (in our case the class of the sample/the phenotype of the recipient post-FMT). In ML, the model usually contains a set of parameters called weights, and the ML trains the model by finding the weights that for which the model is in best agreement with the relation between the input and output in the “Training set.”
- *Training set*. The part of the data used to train the model. The quality of the fit between the input and output data on the training set is not a good measure of the quality of the model, since it may be an “overfit.”
- *Overfitting*. A problem occurring when a model produces good results on data in the training set (usually due to too many parameters) but produces poor results on unseen data.
- *Validation set* is a separate set from the training set that is used to monitor but is not used for the training process. This set can be used to optimize some parts of the learning process including setting the “hyperparameters.”
- *Test set*. Data used to test the model that is not used for either hyperparameter optimization or the training. The accuracy estimated on the test set is the most accurate estimate of the accuracy.
- *Model hyperparameters* are adjustable values that are not considered part of the model itself in that they are not updated during training but still have an impact on the training of the model and its performance. To ensure that those are not fitted to maximize the test set performances, the hyperparameters are optimized using an internal validation set.
- *10-fold cross-validation (referred to as 10 CVs)*―a resampling procedure used to evaluate machine learning models on a limited data sample. The data is first partitioned into 10 equally (or nearly equally) sized segments or folds. Subsequently, 10 iterations of training and validation are performed such that within each iteration a different fold of the data is held-out for validation, while the remaining 9 folds are used for training.
- *Receiver operating characteristic curve (ROC)*―a graph showing the performance of a classification model at all classification thresholds. This curve plots two parameters: true positive rate (TPR―the probability that an actual positive will test positive) and false positive rate (FPR―the probability that an actual negative will test positive).
- *Area under the ROC curve (AUC)*. The (AUC) is a single scalar value that measures the overall performance of a binary classifier. The AUC value is within the range [0.5–1.0], where the minimum value represents the performance of a random classifier and the maximum value would correspond to a perfect classifier (e.g., with a classification error rate equivalent to zero). It measures the area under the ROC curve we define above.
- *Genetic algorithm (GA)*. GA is a method for solving constrained and unconstrained optimization problems that uses iterations of selection and modifications on a family of solutions. Here, we optimize the best-planned transplant (bacterial cocktail) by combining a simple GA model with the iMic predictor.

### Models

We trained our models (described in full in the Supplementary Methods) on all datasets together by zero-padding the missing taxa. Data pre-processing, merging (to the species level), and normalization were performed following the MIPMLP protocol [58] (see Supplementary Methods for a detailed explanation). To identify an ideal donor, we predicted for each recipient (1) the post-FMT Shannon diversity, (2) the frequency of different taxa in post-FMT recipients (at the order and species levels) and binary species presence or absence, and (3) improvement in clinical condition. A specific model was built to predict the relative abundance or prevalence of each taxon separately, considering only donor microbiota characteristics. These models were also validated based on data collected in a human-to-antibiotic-pretreated mouse validation experiment. The same models were applied to 6 clinical human-to-human FMT studies (see the “Experimental datasets” section and Table 3). We trained our model to predict each clinical outcome separately at different time points post-FMT. Furthermore, we developed a mixed model using data from donors to FMT responders and non-responders across all shotgun cohorts [55–57].

### Comparison of donor and recipient microbiome samples

In several cohorts, the same donor stool was given to multiple recipients, resulting in multiple post-FMT recipient properties (e.g., Shannon diversity) for the same donor. To compare the effect of the donor microbiome vs the recipient, we defined the similarity between samples as the Euclidean distance between the MIPMLP [58] preprocessed order frequency of two recipients from the same group or as the difference between the Shannon diversity of two recipient samples from the same group. 3 groups were defined:

- *Same donor - same recipient (SDSR)*. All distances between samples of a single recipient, collected at different time points post-FMT, to measure the temporal variation in a recipient.
- *Same donor - different recipients (SDDR*). All distances between all the different recipients that received FMT from the same donor and were sampled at the same time point post-FMT. This measures the effect of the recipient’s background on its post-FMT microbiome.
- *Different donors - different recipients (DDDR)*. All distances between recipients that received a transplant from different donors, and were sampled at the same time point. This adds the effect of the donor to the effect of the background.

### Validation experiment

The validation experiment consisted of 4 steps:

#### Step A ―Predicting recipient microbiota properties post-FMT

A large cohort of candidate donors *D* was assembled from gut microbiome data previously characterized. Each sample in *D* was passed through the MIPMLP-preprocessing, as above, with the “mean” merge method applied to the species level and a log-normalization. The 7 days post-FMT expected outcome of the transplant with each donor sample was computed using the pre-trained iMic model.

#### Step B―Group definition for the FMT validation experiment

The donor cohort was run through the ideal donor identification model described above, and the donors were divided according to their expected post-FMT microbiome richness: 8 optimal adult donors and 16 sub-optimal donors, 8 of which were children (least optimal) and 8 of which were adults (also sub-optimal and biologically more relevant as donors than children) were selected. Then, a human-to-mouse FMT experiment was performed.

#### Step C―FMT experiment

Briefly, 48 Swiss Webster mice were raised under conventional conditions in the animal facility at the Azrieli Faculty of Medicine, Bar-Ilan University, with controlled temperature (22$^{\circ }$C) and light cycle (12 h light and 12 h dark). The mice had free access to food and water. At 6 weeks of age, they received antibiotics for 2 weeks (ciprofloxacin (0.04 g), metronidazole (0.2 g), and vancomycin (0.1 g)) via their drinking water (400 ml, changed every 3 days). At week 8, after antibiotic treatment, stools were taken, and the mice were randomized into 3 groups (16 mice each). There were 4 cages of male mice for each group (8 mice total) and 4 cages of female mice for each group (8 mice total) for a total of 8 cages (16 mice) per group. After the mice were separated into groups, they were weighed (as a general parameter of recipient state) and then two FMTs were carried out 1 week apart. Cagemates received FMTs from the same human donor. Fecal samples from mice were collected weekly for 6 weeks following FMT, and microbiota were characterized by sequencing the V4 region of the 16S rRNA gene (see Supplementary Methods for details).

We repeated the analysis (not the experiment) by dividing the transplants only from adults based on the expected order frequencies. We only analyzed orders that were predicted with high accuracy (Fig. 3A, step B).

#### Step D―Recipient samples analysis

Following microbiota characterization, recipients’ feces were collected at 8 weeks (8W), 10 weeks (10W), and 15 weeks (15W). DNA was extracted from all mice fecal samples, using the MagMAX Microbiome Ultra-Kit (Thermo Fisher, Waltham, MA) according to the manufacturer’s instructions and following a 2-min bead beating step (BioSpec, Bartlesville, USA). The V4 region of the bacterial 16S rRNA gene was amplified by polymerase chain reaction (PCR) using the 515F (AATGATACGGCGACCACCGAGATCTACACGCT) barcoded and 806R (TATGGTAATTGTGTGYCAGCMGCCGCGGTAA) primers [59] with a final concentration of 0.04% of each primer and 0.5% of PrimeSTAR Max DNA Polymerase (Takara-Clontech, Shiga, Japan) in 50$\mu$l total volume. PCR reactions were carried out by 30–35 cycles of denaturation (95$^{\circ }$C), annealing (55$^{\circ }$C), and extension (72$^{\circ }$C), with final elongation at 72$^{\circ }$C. PCR products were purified using XP magnetic beads (Beckman Coulter, Indianapolis, IN) and quantified using the Picogreen dsDNA quantitation kit (Invitrogen, Carlsbad, CA). Samples were then pooled in equal amounts, loaded on 2% agarose E-Gel (Thermo Fisher, Waltham, MA), purified, and sent for sequencing using the Illumina MiSeq platform (Genomic Center, Azrieli Faculty of Medicine, Bar-Ilan University, Israel). Microbiota properties (Shannon diversity and orders’ frequency) were calculated at 1 week (referred to as 10W) post-FMT and 6 weeks (referred to as 15W), post-FMT.

### Synthetic community compilation by generative GA

To identify an ideal synthetic community for microbiota manipulation that would increase the richness and have a high probability of engraftment, we used a GA [60] to first identify donors that would result in an optimal recipient outcome (microbial richness or specific bacterial order engraftment). To this end, we simulated 2083 donor profiles from the actual donor cohort data, as described in the Supplementary Methods. Then, 100 donors were randomly selected, and we modeled the predicted richness of recipients of FMTs from the simulated donors, as described in the Supplementary Methods. The aim of the model was to generate the optimal synthetic community for a given outcome, while minimizing non-zero taxa, though no minimum number of taxa was required. The 30 donors with the highest loss in the maximization task and the 30 donors with the lowest loss in the minimization task were then chosen for the creation of the next generation (Fig. 6A, step D), namely the creation of an optimal synthetic microbiota. In the next generation, the same process was performed, and to enrich the variability of children, we added mutations and recombination with a probability of 0.3 (see Supplementary Methods). Then, synthetic FMT performance was again tested, and ideal “parents” were again selected. This generative process of creating simulated microbiota continued for 25 generations, as we found convergence to occur after this many iterations. Details of GA modeling considerations and methods are presented in the Supplementary Methods.

### Statistics and validation

#### R2 score

To evaluate the performance of our predictors, we calculated the R2 metric on external test sets using 10 cross-validations (CVs). We reported the average R2 score of the 10 runs. Then, we applied an ANOVA test to check whether the performances of the models were significantly different. If ANOVAs were significant, we used one-sided *T*-tests for pairwise comparisons.

#### Spearman correlation coefficient (SCC)

To evaluate the performance of our predictors, we calculated the SCC metric on the external test set using 10 CVs. We reported the average SCC of the 10 runs. Then, we applied an ANOVA test to check whether the performances of the models were significantly different. If the ANOVA were significant, we kept comparing by a one-sided *T*-test.

#### Area under the ROC curve (AUC)

To evaluate the performance of our predictors on the absence-presence species task, we calculated the AUC metric on the external test set. Then we applied a one-sided *T*-test between the RF (random forest) model and the iMic model.

#### Biological relevance test

To assess the biological relevance of our donor-picking algorithm (according to the predicted Shannon diversity), we compared the predicted recipient richness of the clinically defined FMT “successes” and “failures” by applying a two-sided *T*-test.

#### Same donor condition distribution

In a second scenario, we examined the actual distribution of conditions based on donor strain colonization in recipients undergoing FMT. To analyze this distribution, we utilized data from the 5 aforementioned conditions as well as additional data from the study conducted by Schmidt et al. [61]. For each donor, we assessed the number of successful transplants among all the transplants associated with that donor. This analysis provided insights into the efficacy and variability of FMT outcomes.

## Results

### FMT studies

**Fig. 1:**
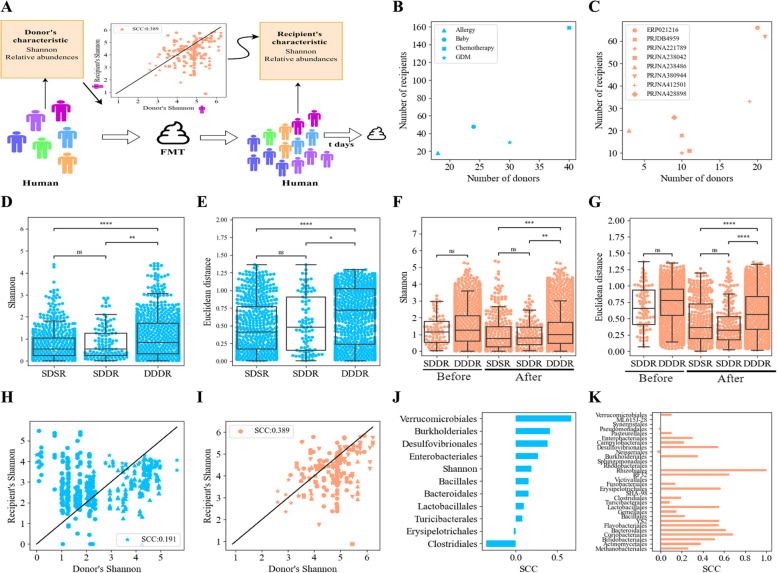
Relations between donor and recipient microbiomes. Note along the figure light blue represents analyses based on the human-to-GF cohorts, and light orange represents analyses based on the human-to-human cohorts. **A** Schematic figure of the raw relations between the donor’s samples properties and the recipient’s samples post-FMT treatment. The properties we followed during this analysis were the Shannon, the order’s relative abundances, and the species relative abundances and presence or absence. Each color represents a cohort. The raw relations were measured by calculating the SCC between the property in a donor sample and a recipient sample. **B** Scatter plot of the number of donors vs the number of recipients in each cohort over the human-to-GF cohorts, where GDM was marked with an asterisk (*), Allergy was represented by a triangle symbol, Chemotherapy was marked with an X, and Baby with a dot. **C** Scatter plot of the number of donors vs the number of recipients in each cohort over the human-to-human cohorts. **D**, **E** Similarity Shannon differences (**D**) and Euclidean distance between two recipients preprocessed ASVs (order-level) vectors in the human-to-GF cohorts (**E**). The rightmost bar, SDSR, represents the distances between samples of the same recipient (GF mouse) and the same donor (measures the time variability); the middle bar represents the distances between samples of different recipients (GF mice) that got FMT from the same donor, SDDR (measures the effect of the recipient background); and the leftmost bar represents the distances between samples of different recipients (GF mice) that received FMT from different donors, DDDR (measures the effect of the donor), with a significant hierarchy of distances. The lowest distances are within the same donor/recipient, followed by the same donor, followed by different donors. **F**, **G** Similarity Shannon differences (**F**) and Euclidean distance between two recipients’ preprocessed ASVs (order-level) vectors in the human-to-human cohorts (**G**) before and post-FMT. The rightmost bar represents the distances between samples of the same mouse and the same donor (SDSR, samples along time), the middle bar represents the distances between samples of different mice that got FMT from the same donor (SDDR), and the leftmost bar represents the distances between samples of different mice that received FMT from different donors (DDDR), with a clear and significant hierarchy of distances. The lowest distances are the same donor/recipient, followed by the same donor, followed by different donors ($$*$$ $$p<0.05$$, $$**$$ $$p<0.01$$, $$***$$ $$p<0.001$$). When comparing the results before the transplant, there is no difference between the groups (before in plots **F** and **G**). **H**, **I** Scatter plots of donor’s Shannon vs recipient’s Shannon in human-to-GF cohorts (**H**) and human-to-human cohorts (**I**). The black line represents the $$y=x$$ line of a perfect match between the donor and recipient properties and each shape represents a different dataset according to the shapes in **B** and **C**. Similar results for all the orders are in Supplementary Material Figs. S4 and S5. **J**, **K** All donor-recipient orders and Shannon diversity SCCs in the human-to-GF cohorts (**J**) and the human-to-human cohorts (**K**)

To investigate the relationship between the donor microbiome and FMT outcomes, we conducted an analysis of transplants involving the transfer of stool samples from human donors to either GF mice or humans across 12 different cohorts (Fig. 1A). To ensure our findings are general enough, we combined data from multiple experiments within each cohort separately (i.e., human-to-GF and human-to-human cohorts). In each experiment, we analyzed recipient stool samples several weeks post-FMT, as well as from the human donors. Both the recipient and human donor samples underwent 16S sequencing (see the “Methods” and Supplementary Methods for sequencing, pre-processing, and combination of samples).

For the human-to-GF cohorts, we analyzed 4 cohorts (Table 1). The first cohort, denoted as Gestational Diabetes Mellitus (GDM), was previously reported by Pinto et al. [12]. The second cohort consisted of stool samples from patients with IgE-mediated food allergies, designated as Allergy [46]. The third cohort involved a chemotherapy experiment referred to as Chemotherapy [15]. Lastly, we included a cohort that examined the long-term impact of antibiotic treatment during the neonatal period and early childhood on child growth, denoted as Baby [13].

We also analyzed 8 human-to-human cohorts [47–54] (Table 2). In some experiments, the recipients received antibiotic treatment (ABX), which included the following projects: ERP021216, PRJNA221789, PRJNA238042, PRJNA238486, PRJNA380944, and PRJNA412501. In contrast, other experiments involved recipients who did not undergo antibiotic treatment, such as PRJDB4959 and PRJNA428898.

Given that various transplant experiments employed different outcome measurements, we initially focused on identifying outcomes that were shared between experiments and could be generalized across multiple scenarios. For instance, we examined the engraftment success by assessing post-FMT recipient Shannon diversity, as well as the post-FMT relative abundances of different taxa at the order or species level and their binary presence or absence. We then further directly studied the clinical impact of the FMT.

### Relation between donor and recipient microbiome properties

We first tested for a relation between the donor and recipient microbiome. In several cohorts (e.g., the Baby cohort and all the human-to-human cohorts), the same transplant (stool of the same donor) was applied to multiple recipients (Fig. 1B, C). These recipients may have different properties (e.g., Shannon diversity or orders relative abundances). To compare the effect of the donor microbiome on the post-FMT microbiome, we defined the similarity between samples as the Euclidean distance between the MIPMLP preprocessed order frequency of two recipients from the same group or as the difference between the Shannon of two recipients samples from the same group.

3 groups were defined:

- *Different time points within the same recipient (SDSR)*. Distances between the samples of a certain recipient at different time points post-FMT to measure the temporal variation in a recipient.
- *Same donor - different recipients (SDDR)*. Distances between all the different recipients that received the FMT from the same donor at the same time point. This measures the effect of the recipient’s background on its microbiome.
- *Different donors - different recipients (DDDR)*. Distances between recipients that received a transplant from different donors at the same time point. This adds the effect of the donor to the effect of the background.

In the human-to-GF cohorts, there was no initial recipient microbiome. However, the recipient may have microbiome-independent factors affecting the outcome. In the human-to-human cohorts, to ensure that we really study the effect of recipient background vs donor microbiome and not basal differences in human recipients, we again compared the distances between the SDDR group and the DDDR group of the recipient after antibiotic treatment and before the FMT and there were no significant differences (Fig. 1F and G “Before”). In both the human-to-GF and human-to-human cohorts and in the Shannon diversity and relative abundance differences, there were no significant differences between the SDSR and SDDR groups (Fig. 1D, E and F, G “After”).

However, the differences were significantly lower in the groups with FMT from the same donor (SDSR and SDDR) than in all other groups (Fig. 1D, E and F, G “After” one-sided *T*-test with *p*-value $$< 0.05$$ for the composition and *p*-value $$< 0.01$$ for the Shannon in the human-to-GF cohorts and (*p*-value $$< 0.01)$$ for the Shannon or (*p*-value $$< 0.0001)$$ for the compositions in the human-to-human cohorts). To summarize, the donor’s microbiome influence is stronger than the recipient’s background, even in human recipients that exhibit a diverse initial microbiome.

Given the effect of the FMT properties on the recipient, one may suggest that the donor and recipient taxa compositions are highly similar, and as such donors that have the maximal frequency of a given order or a high diversity should be chosen to reach the same goal in the recipient. To examine the relations between donor and recipient properties, we computed the SCC between the Shannon diversity (distribution information can be found at Supplementary Material Fig. S1A and C) or the order relative frequencies of the donors and the recipients post-FMT (distribution information can be found at Supplementary Material Fig. S1B and D and S2-S3). In the human-to-GF cohorts, SCCs were low for all properties $(|SCC| < 0.4)$ except for the relative abundance of the Verrucomicrobiales order (Fig. 1H and J and Supplementary Material Fig. S2). The SCCs were higher in the human-to-human cohorts than in the human-to-GF cohorts with an average SCC of 0.439 (Fig. 1J, K), as expected given the similar host. One can thus infer (except for the Verrucomicrobiales and some orders in the human-to-human cohorts) that the post-FMT recipient microbiome is significantly different than the donor microbiome.

Given the low correlations observed between donor and post-FMT recipient microbiomes but the significant connection between them, more advanced algorithms are required to predict transplant microbiome properties based on FMT composition.

### Prediction of recipient post-FMT microbiome properties from donor microbiome

To test whether post-FMT engraftment success, measured by microbiome properties (Shannon diversity, orders and species frequency, and species presence) can be predicted using the donor sample composition, we tested 7 different multivariate predictors using the donor preprocessed ASVs (see experimental setup in Supplementary Methods) as concatenated with the number of days post-FMT as an input to these models to predict different microbiome properties of the recipient post-FMT such as Shannon diversity, 10 (human-to-GF) or 30 (human-to-human) different orders’ relative abundances, 50 (human-to-GF) or 100 (human-to-human) most frequent species relative abundances, and their binary presence or absence.

**Fig. 2:**
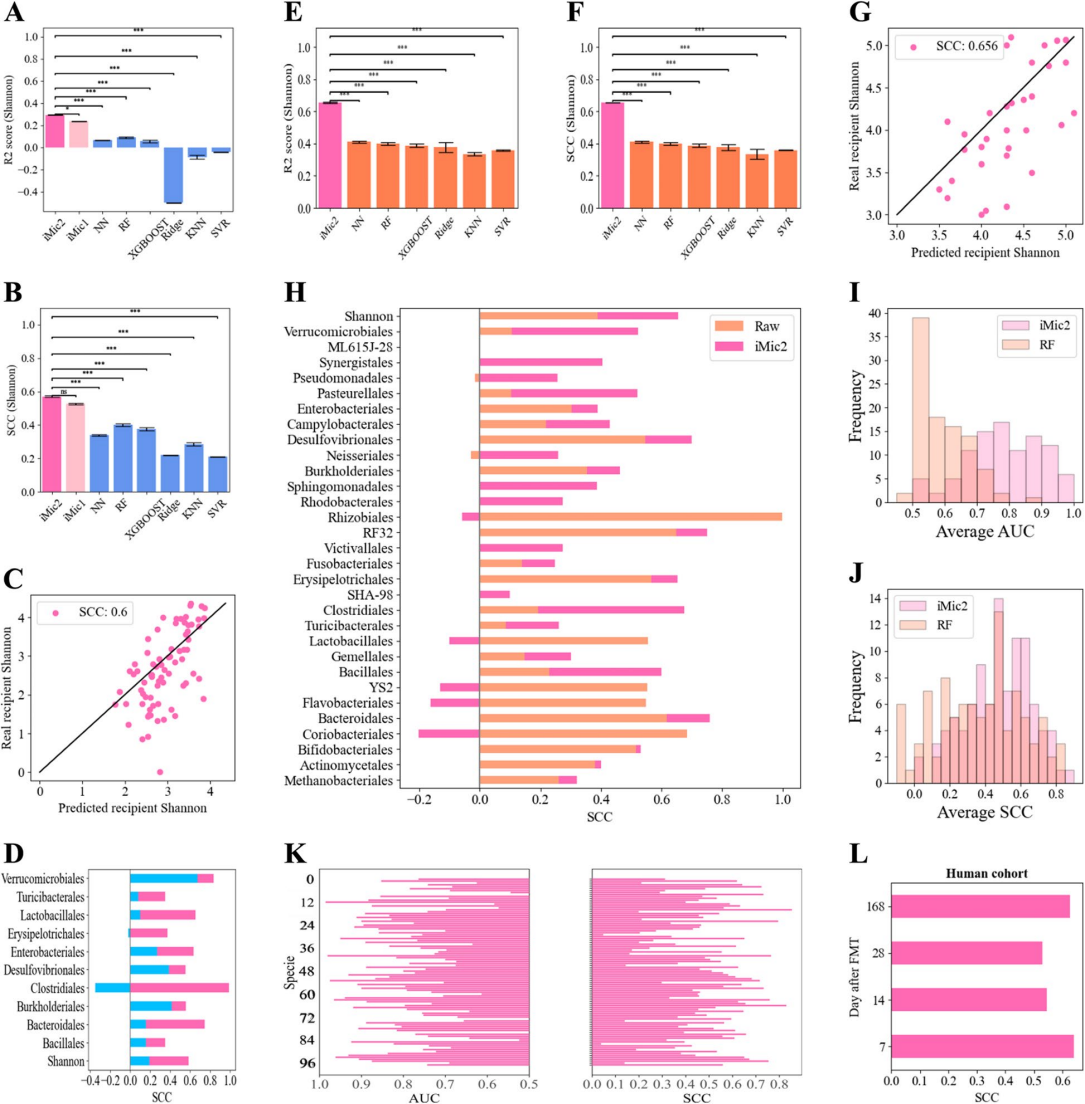
Prediction of recipient post-FMT microbiome properties from donor samples. **A**, **B** Different models evaluations scores of recipients’ Shannon, R2 scores (**A**), and SCC (**B**) over the human-to-GF cohorts (for parallel results on the different orders see Supplementary Material Fig. S7). The *x*-axis represents the model. The simplest models Ridge, KNN, and SVR are in blue; the networks and trees, RF, XGBOOST, and NN are in blue; the structure-based CNNs,  iMic1 and iMic2, are in pink. The standard errors over the 10 CVs are in black. iMic2 outperforms all the other models and predicts the recipient Shannon diversity with R2 of 0.358 and SCC of 0.6. **C** Scatter plot of the recipients’ predicted Shannon after FMT vs the real recipients’ Shannon over the human-to-GF cohorts. The black line represents the $y=x$ line of a perfect match between the recipients’ predicted and real properties. **D** All predicted-real recipients’ properties SCCs over the human-to-GF cohorts, where the raw correlation between the recipient’s property and the donor’s property is in light blue and iMic’s improvement is in pink. **E**, **F** Different models evaluations scores of recipient’s Shannon diversity, R2 scores (**E**), and SCC (**F**) over the human-to-human cohorts (for parallel results on the different orders see Supplementary Material Fig. S9). The *x*-axis represents the model. The simplest models Ridge, KNN, and SVR are in orange; the networks and trees, XGBOOST, RF, and NN, are in orange; and the structure-based iMic2 is in pink. The standard errors over the 10CVs are in black. iMic2 outperforms all the other models and predicts the recipient Shannon diversity with R2 of 0.369 and SCC of 0.656. **G** Scatter plot of the recipients’ predicted Shannon post-FMT vs the real recipients’ Shannon diversity over the human-to-human cohorts. The black line represents the $y=x$ line of a perfect match between the recipients’ predicted and real properties. **H** All predicted-real recipients’ properties SCCs over the human-to-human cohorts, where the raw correlation between the recipient’s property and the donor’s property is in orange and iMic’s improvement is in pink. **I**, **J** Histograms of AUCs of presence absence predictions of the species over the human-to-human cohorts (**I**) and of SCCs of the compositions predictions over the human-to-human cohorts (**J**). The *x*-axes represent the bins of scores, AUC (**I**)and SCCs (**J**), and the *y*-axes represent the number of different taxa that got that score. For parallel results on the human-to-GF cohorts see Supplementary Material Fig. S6**K** AUCs and SCCs of the 100 most frequent species. The left *x*-axis represents the presence or absence AUC over the human-to-human cohorts (pink), while the right *x*-axis represents the SCC (black). For parallel results on the human-to-GF cohorts, see Supplementary Material Fig. S6. **L** The prediction remains accurate long after the transplant in the human-to-human cohorts. The *x*-axis represents the SCC, and the *y*-axis represents the number of days post-FMT. There is not a significant difference in the SCCs over the time followed

The models tested included simple models such as a K-nearest neighbors regression (KNN), support vector machine regression (SVR), and Ridge regression that gave quite poor results similar to the low SCCs in the univariate analysis $$(SCC < 0.4)$$, with extremely low R2 scores (Fig. 2A, B). More complex models tested include random forest (RF), XGBOOST, and a fully connected neural network (NN). These models’ predictions were more accurate than the simple models’ predictions. The highest SCC was obtained by applying the iMic model [62] to the donors’ MIPMLP preprocessed taxa frequencies, with an SCC of 0.6 +/− 0.004 and an R2 score of 0.358 +/− 0.003 on the Shannon diversity (Fig. 2A, B pink bars). The results are similar on the order relative abundance with an average SCC over all orders of 0.568 (Supplementary Material Fig. S6). These SCCs and R2s are much higher (*p*-value $$< 0.0001)$$ than the ones obtained from the univariate relations (Figs. 1H vs 2C) on the Shannon diversity as well as on all the other properties (Fig. 2D).

Similar results were obtained when predicting species’ relative abundances instead of orders. We predicted for the 50 most frequent species both the presence or absence of the species from the recipient samples 1-week post-FMT (in such cases the average AUC is reported over 10 CVs on the appropriate external test set) and their relative abundances (in such a case the SCC was similarly reported). The average AUC of all the species was 0.8 +/− 0.11. For the species relative abundance predictions; the average SCC over 10 CVs of all the species was 0.52 +/− 0.16 (Supplementary Material Fig. S4A). An RF (which was the best model after iMic) was also applied to these tasks to compare to existing methods [35]. Histograms of the RF results vs iMic can be found in Supplementary Material Fig. S4B, C. iMic is significantly better than the RF model (*p*-value $$< 0.001)$$.

The results above were from models trained on a mixture of datasets (human-to-GF) to ensure that the results are not an artifact of a single experimental setup. To test the prediction accuracy between datasets, we ran iMic on a leave-one-dataset-out (LODO), where the whole Chemotherapy dataset (which contains the least number of samples) was not used during the training of the model. The model was trained on the merged 3 cohorts and was tested on the Chemotherapy dataset. The prediction accuracy was slightly lower than the mixed learning prediction, but much better than the univariate natural correlations (Supplementary Material Fig. S8).

The post-FMT microbiome properties predictions are further improved by including the donor’s age, sex, and weight, when available (see Supplementary Methods for data completion). When the donor metadata is used, the AUC obtained using donor-only data reaches similar values to the ones reported using both donor and recipient properties (Supplementary Material Fig. S4D [35] and for the contribution of the metadata to the predictions Supplementary Material Fig. S5)―a Pearson correlation of 0.7 for the Shannon and an AUC of 0.85 for the presence-absences predictions.

We used the same model and same hyperparameters as for the mouse and trained a model to predict human post-FMT microbiome properties (for hyperparameters used see Supplementary Material Table S1-S3). Again, all the samples of the same recipient were assigned to the same group to prevent data leakage. iMic significantly (*p*-value $< 0.001$) best predicted the recipient’s post-FMT Shannon diversity(R2 = 0.369 +/− 0.001 and SCC = 0.656 +/− 0.005, Fig. 2E–G). The SCC values of iMic were much higher than the direct correlation between donor and recipient both on the Shannon and the order relative abundances (Fig. 2H). Similar results were obtained when predicting orders relative abundances (Fig. 2H), the presence or absence of the recipient species (Fig. 2I and left K), and the SCC of the recipient species compositions (Fig. 2J and right K). In contrast to the short-term effect of the human-to-mouse FMT (Fig. 5E, F), the effect of the transplant and the prediction accuracy did not decrease even 6 months post-FMT (Fig. 2L).

**Fig. 3:**
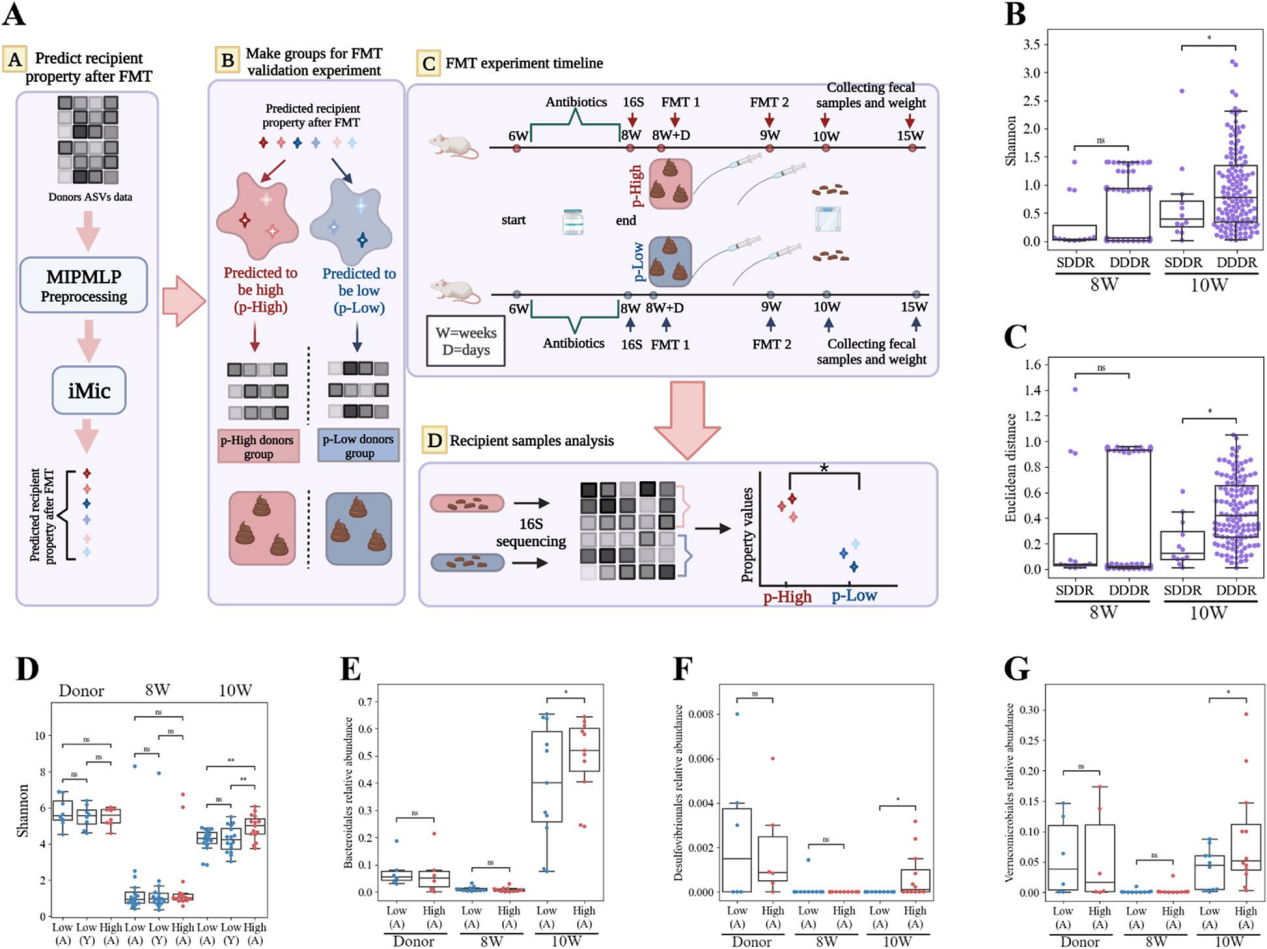
Validation experiment. **A** Validation experiment’s schematic figure. Step A―Predicting the recipient’s property post-FMT. Inserting all the MIPMLP preprocessed donors from all the cohorts (including donors that had not been actually transplanted) into the pre-trained iMic model, using existing datasets. iMic returns the predicted recipients’ properties post-FMT. Step B―Grouping samples for FMT validation experiments. Two groups were defined from the predicted properties. A group of predicted to be high (with predicted high values of the property) and predicted to be low (with predicted low values of the property). Step C―FMT experiment timeline. Two groups of mice were raised till the age of 6 weeks. They got antibiotic treatment (weeks 6–8). At the age of 8 weeks, stools were collected and sequenced. At the age of 8 weeks + 1 day, they received the first FMT. One group of mice got the FMT from the predicted to be high donors group, and the second group of mice got the FMT from the predicted to be low group. They got the second FMT from the same donors at the age of 9 weeks. Stool samples were collected a week after the second transplant at the age of 10 weeks and 6 weeks after the second FMT treatment at the age of 15 weeks. Step D―Recipient samples analysis. The real recipients’ properties were calculated from the mice’s stool samples at the age of 10 weeks. A comparison between the 2 groups’ properties showed significant differences in the targeted properties. **B**, **C**  Difference between Shannon diversities (**B**) and Euclidean distance between two recipients preprocessed ASVs (order-level) vectors (**C**). The right bar represents the distances between samples of different recipients (ABX mice) that got FMT from the same donor, SDDR, and the left bar represents the distances between samples of different recipients (ABX mice) that received FMT from different donors, DDDR. There is a significant difference between same and different donors post-FMT (10W), but not before (8W) ($$*$$ $$p<0.05$$, $$**$$ $$p<0.01$$, $$***$$ $$p<0.001$$). **D–G** Differences between the donors and real recipients at different time points (8W, 10W) properties on the groups we defined, Shannon (**D**), Bacteroidales order relative abundances (**E**), Desulfovibrionales order relative abundances (**F**), and Verrucomicrobiales order relative abundances (**G**). The (A) represents a transplant from an adult human, while (Y) represents a transplant from a young human. Again, there is no difference before the FMT (8W) or in the donors (Donor)

To validate the accuracy of the predictor developed on retrospective data, we performed a prospective in vivo validation experiment (Fig. 3A). The validation experiment was based on a set of existing microbiome samples, with the following stages (see Supplementary Methods for details):

- Produce a set of candidate donors and predict for each sample in the set the expected outcome (e.g., the expected Shannon―Fig. 3A, step A).
- Separate samples into the ones predicted to induce a high diversity and the ones predicted to induce a low diversity. Since almost all the donors who were predicted to induce a low Shannon were children, we added a group of samples predicted to induce a low diversity aged matched to the samples predicted to have a high diversity (Fig. 3A, step B).
- Perform FMT on 3 groups of antibiotic-treated 8-week-old mice (see the “Methods” section) from the 3 groups of donors above, and collect stool samples at the age of 10 weeks (see the “Methods” section, Fig. 3A, step C).

We first checked for the effect of the donor on the transplant in antibiotic-treated mice. Indeed, there is a significant difference (*p*-value $$< 0.05)$$, between the SDDR group and the DDDR group in the post-FMT recipients (referred to as 10W, Fig. 3B, C), and no significant difference between these groups after antibiotic treatment and before the FMT (referred as 8W, Fig. 3B, C).

We then tested the quality of the prediction by comparing the difference between the Shannon diversity from the groups predicted to have low and high post-FMT diversities (*p*-value $< 0.01$). There were no significant differences between the predicted to be low groups as a function of the donor’s origin (adult vs child, Fig. 3D).

We then applied a set of similar experiments, where we used the same samples above (only adults to avoid the effect of the donor age), but defined the “high” and “low” groups according to the expected relative abundances of different orders as computed by iMic for all the orders properly predicted in the initial analysis (the orders where the initial AUC of iMic was higher than 0.5). Again, the recipients’ order frequencies were measured in the mice stool samples at the age of 10 weeks. For all orders except for the Enterobacterials, the groups predicted to have a higher frequency for the appropriate order indeed had a higher frequency ($p< 0.05$ for Bacteroidales, Desulfovibrionales, and Verrucomicrobiales) (Fig. 3A, step D, D–G). In general, Gram-negative bacteria were well predicted in contrast to Gram-positive bacteria. Similar results were recently demonstrated by Ianiro et al. [35].

To ensure that the difference is not the result of the recipient microbiome (after the antibiotics treatment), we repeated the analysis on the samples after antibiotic treatment and before FMT (Fig. 3D–G 8W), with no difference between the groups (Fig. 3D–G 8W). To test that the difference in the recipient is not only a mirror of the differences in the donors, we compared the donor samples’ microbiome properties, with again no difference between the groups (Fig. 3D–G Donor).

### Prediction generalization to clinical contexts

Following the prediction of the post-FMT microbiome properties, we checked whether the FMT clinical outcome can be predicted using only the donor microbiome. In this context, one must separate transplants in recipients with CDI and transplants in non-CDI recipients. In CDI FMT, there is a very high success fraction, so there is almost no need for an outcome prediction model [63, 64]. We thus developed models for the post-FMT non-CDI clinical outcomes. We tested various clinical conditions, such as IBD, IBS (irritable bowel syndrome), melanoma, UC, and antibiotic resistance [37, 47, 55–57, 61].

**Fig. 4:**
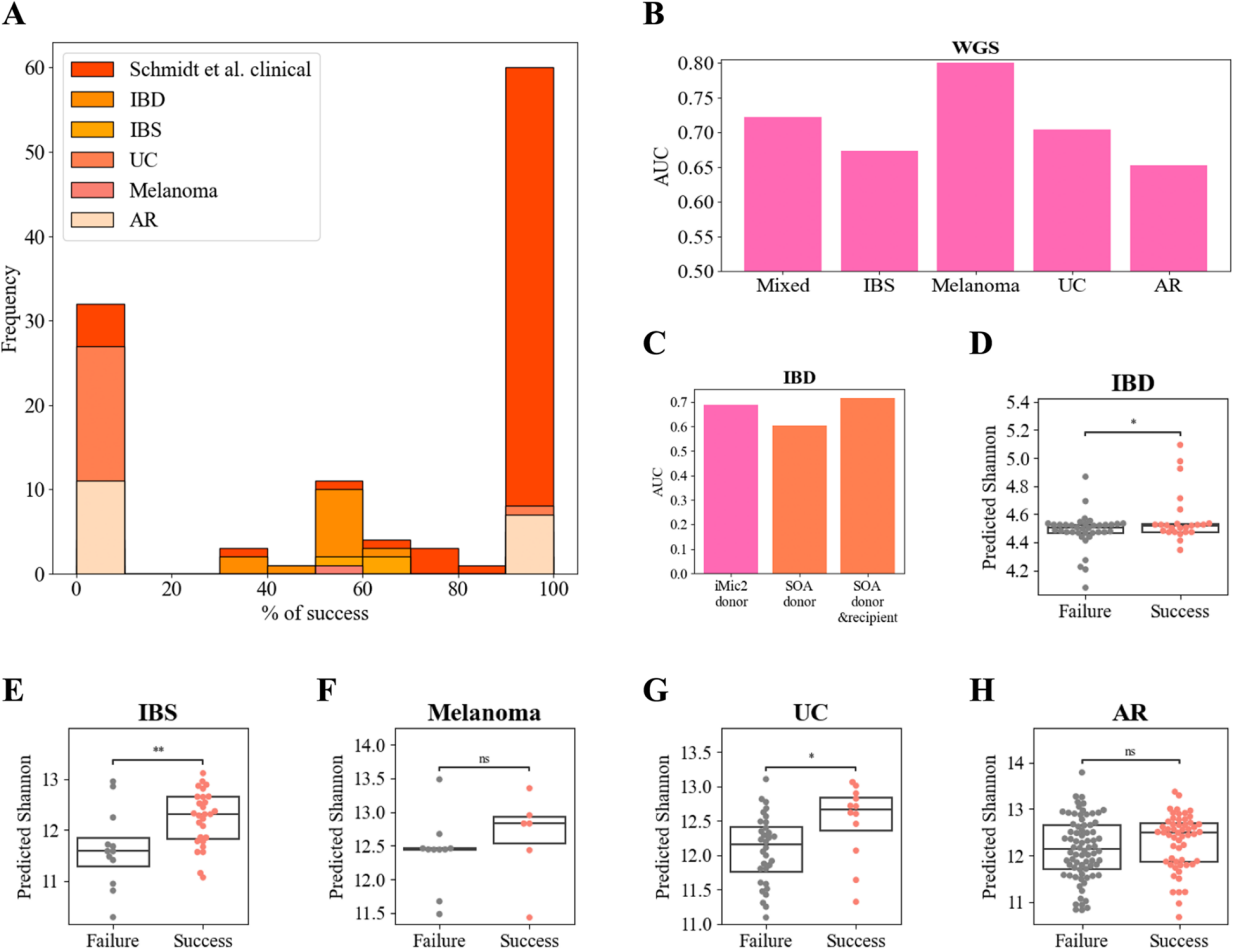
Clinical predictions post-FMT and engraftment success vs improvement in recipients clinical symptoms post-FMT. **A** Distribution of success vs failure given a certain donor over 6 clinical cohorts. The *x*-axis represents the percent of success given a certain donor, the *y*-axis represents the frequency. **B**, **C** iMic predictions of improvement in recipients’ clinical symptoms in WGS cohorts (**B**) and IBD 16S cohort (**C**). In the IBD cohort, we also compare our predictions to state-of-the-art (SOA) reported predictions in [37] **D–H** Swarm plots of predicted recipients’ Shannon of the subjects their FMT succeeded and the subjects their FMT failed over different clinical conditions IBD (**D**), IBS (**E**), melanoma (**F**), UC (**G**), and antibiotics resistance (AR) (**H**). A two-sided *T*-test was applied between the success and failure groups of each cohort ($$*$$ $$p<0.05$$,$$**$$ $$p<0.01$$, $$***$$ $$p<0.001$$)

We first tested if the outcome was mainly determined by the donor properties. If that would be the case, all recipients receiving transplants from a particular donor would either consistently succeed or fail. We calculated the fraction of recipients for each donor group where the treatment succeeded. The results varied among conditions. For instance, colonization success (in the Schmidette et al. cohorts), melanoma response to PD-1 therapy, and antibiotic resistance exhibited strong donor consistency, whereas other conditions showed very limited consistency (Fig. 4A).

We further employed the iMic model to predict the clinical conditions of recipients across various datasets, including WGS cohorts (AUC   0.71, Fig. 4B) and the 16S cohort (AUC = 0.689, Fig. 4C). In the IBD cohort, where previous predictions were published [37], we compared our results with the state-of-the-art donor-based model (AUC = 0.605), recipient-based learning (AUC = 0.706, similar to iMic’s donor model), and a combined donor and recipient model (AUC = 0.716). Thus, not only can post-FMT microbial properties be predicted from the donor but also the clinical outcome.

### Engraftment success vs improvement in recipients post-FMT clinical symptoms

To assess whether an “ideal donor” defined by its richness (Shannon diversity) accurately predicts improvement in clinical symptoms, we compared the predicted recipient post-FMT richness and the improvement in clinical outcome (“Success”) in different cohorts. The model consistently predicted higher Shannon diversity in all conditions for the group where the clinical outcome improved (significant $p-value < 0.05$ in 3 out of 5 cases, Fig. 4D–H).

### Recipient effect on the post-FMT predictions

To better understand the effect of the recipient on the prediction of the transplant outcome, we compared 4 levels of recipient diversity:

1. GF mice― no recipient initial microbiome, and all mice grow in similar conditions.
2. Antibiotic treated mice (ABX)―most of the recipient’s initial microbiome is destroyed and all mice grow in similar conditions.
3. Antibiotic treated human (ABX)―most of the recipient’s initial microbiome is destroyed, but the recipients live in different conditions.
4. Humans, with no antibiotic treatment―the recipient’s initial microbiome is intact, and the recipients live in different conditions.

**Fig. 5:**
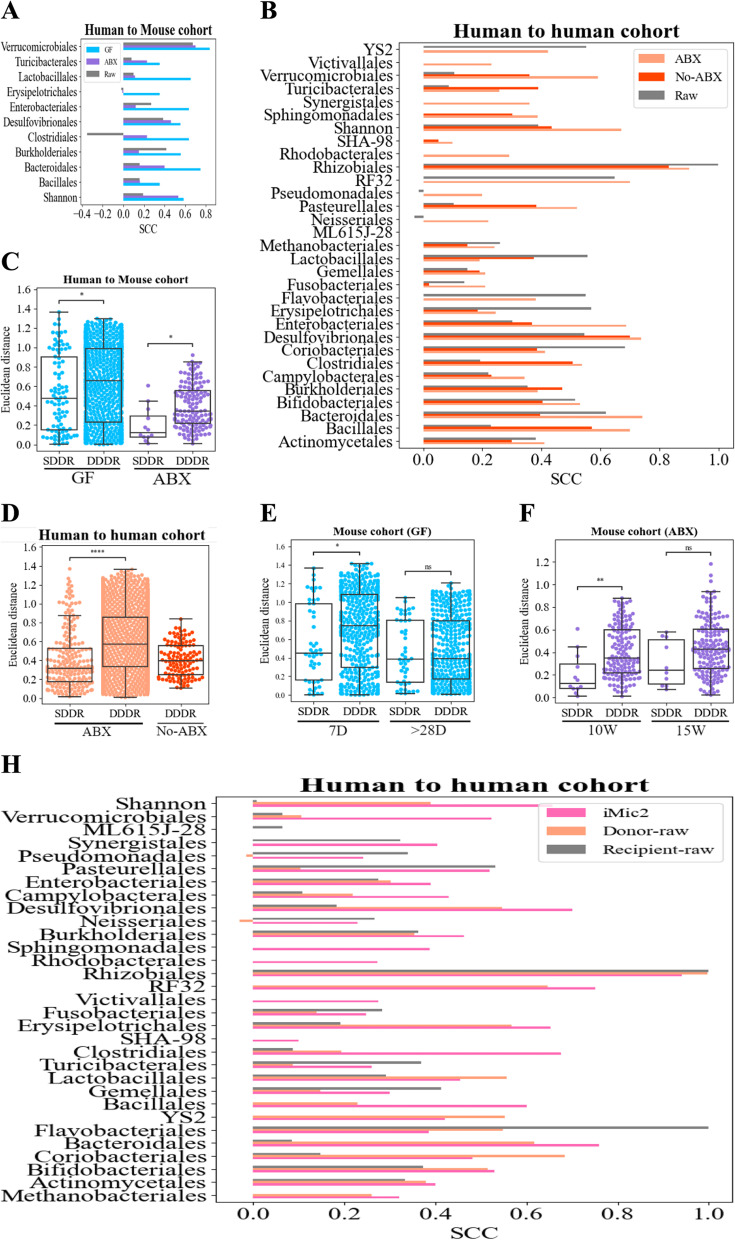
Recipient effect on post-FMT predictions. **A** SCCs of different orders and Shannon reported over the ABX-treated mice (purple bars) vs the GF mice (light blue bars) compared to the overall raw donor-recipient correlations (gray).**B** SCCs of different orders and Shannon reported over the ABX treated cohorts (light bars) vs the no-ABX, untreated cohorts (dark bars) compared to the overall raw donor-recipient correlations (gray). **C**, **D** Similarity Euclidean distance between two recipients preprocessed ASVs (order-level) vectors in the human-to-mouse cohorts (GF and ABX) (**D**) and the human-to-human cohorts (ABX vs no ABX). In each pair, the rightmost bar represents the distances between samples of different recipients that got FMT from the same donor , SDDR (measures the effect of the recipient background), and the leftmost bar represents the distances between samples of different recipients that received FMT from different donors, DDDR (measures the effect of the donor), with a significant hierarchy of distances. The lowest distances are within the same donor/recipient, followed by the same donor, followed by different donors ($$*$$ $$p<0.05$$,$$**$$ $$p<0.01$$, $$***$$ $$p<0.001$$). **E**, **F** Comparison of FMT effect in the mouse cohorts in GF mice (**E**) and in ABX mice (**F**) at different times. In the GF mice, there is a donor effect at 7D and not at 28D. Similarly, in the ABX-treated cohort, there is a difference at 10W (1W post-FMT) and not at 15W (6 weeks post-FMT). **G** Comparison of SCCs between the recipient’s properties post-FMT with the donor (orange), the recipient before the FMT (gray), and the predicted recipient property post-FMT by iMic (pink) in the human-to-human cohorts. The prediction is typically much higher than the two others

iMic managed to predict the recipient post-FMT outcome in the 4 groups with decreasing accuracy as the recipient microbiome becomes more important (Fig. 5A and B). However, even in untreated humans, the prediction is much better than just using the donor as a prediction (Fig. 5B). Similarly, the difference between SDDR and DDDR groups decreases as the recipient microbiome becomes more and more important (Fig. 5C and D). We did not perform this comparison with the group of human recipients with no antibiotics, since in this group each recipient had a different donor.

### Optimal artificial mixture of grown microbes

The results above on both human and mouse recipients highlight the possibility of choosing among multiple candidate donors. However, for an optimal outcome, one may want to develop de-novo transplants. One can propose, for example, an artificial planned transplant to promote a specific taxon. As mentioned, transplanting a given taxon will not always increase its abundance post-transplant. As such, a mixture of microbes is required. A complex transplant containing a large number of taxa can be generated to produce the required outcome. However, the number of taxa that can be used in such a mixture is limited. Therefore, a balance between the number of taxa that are needed to be generated for the FMT and the target outcome is required.

**Fig. 6:**
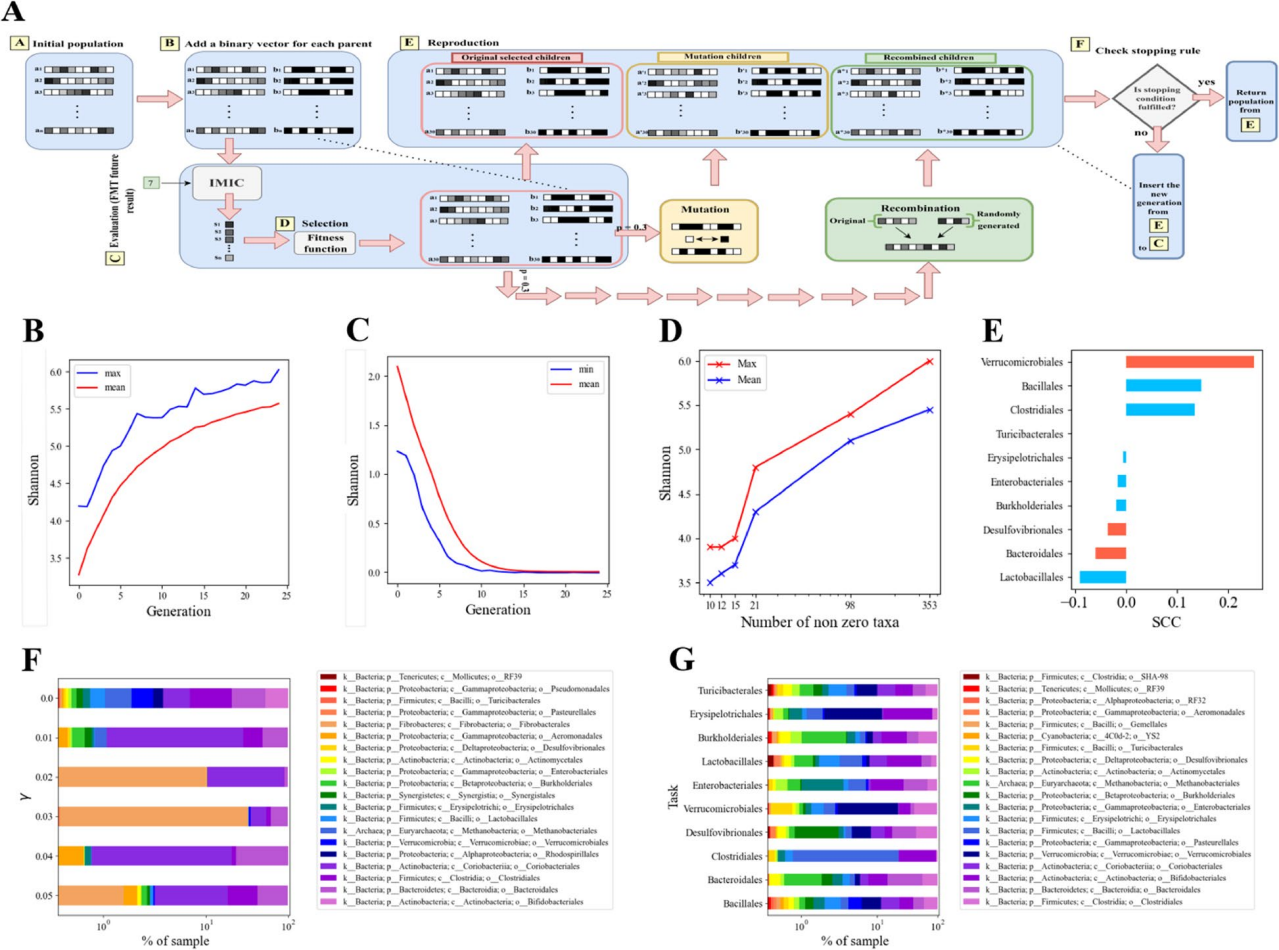
Optimal artifical mixture of grown microbes A GA schematic figure. The GA contains the following steps: Step A―Initial population. One hundred donor samples are randomly sampled from the donors of all the cohorts. Step B―Adding a binary vector to each parent donor. The binary vector consists of 1 when the ASV’s abundance is higher than 0, and is 0 otherwise. Step C―Evaluation of recipients’ FMT future result after a week. By applying the pre-trained iMic model to the parent donors, we get the future recipients’ outcomes. Step D―Selection. The selection is done according to our fitness function choosing the best 30 donors with the most appropriate recipients outcome. Step E―Reproduction. To complete the parents of the next generation a mutation occurs with a probability of 0.3, and recombination occurs with a probability of 0.3. Step F―Checking stopping rule. If the stopping criterion is met, the donors of step E are returned; otherwise, the new generation of donors from step E is again used for the outcome prediction using iMic’s in C, until the stopping criterion is met. **B**, **C** GA convergences within 25 epochs on the Shannon diversity optimization task for both maximizing (**B**) and minimizing the recipient’s Shannon diversity a week post-FMT (**C**). **D** Monitoring the number of non-zero taxa of donors during the maximizing optimization. The *x*-axis represents the number of non-zero taxa (log scale) and the *y*-axis represents the predicted Shannon diversity of the best donors. **E** SCCs between the property in the optimized donors and the predicted recipient. The significantly predicted orders from the validation experiment are in red. **F**, **G** Percentage of the most common taxa in the optimized donors for the Shannon diversity task for different $\gamma$ values (**F**) and for different prediction tasks (**G**)

To find the optimal required FMT given a targeted outcome, such as: maximizing the recipients’ Shannon, minimizing the recipients’ Shannon, or maximizing the relative abundances of a certain order in the recipients’ samples, we developed a GA. In short, 100 parent donors were randomly chosen from all the donors’ populations in all the cohorts studied - $a_i$ (Fig. 6A, step A). A binary representation, $b_i$, of the MIPMLP preprocessed donor vectors, $a_i$, was created for each donor (Fig. 6A step B). Each MIPMLP preprocessed donor vector, $a_i$, was the input of the pre-trained iMic model and the expected diversity after 7 days was predicted (or any other outcome as discussed above) (Fig. 6 A, step C). All the predicted outcomes, $s_i$, were the input of the following fitness function for the selection of the next generation:

1 $$\begin{aligned} fitness_{max}(s_i,b_i) = s_i - sum(b_i) \cdot \gamma , \end{aligned}$$

such that $sum(b_i)$ represents the number of non-zero taxa in the donor sample, and $\gamma$ is a hyperparameter that controls the importance of the number of non-zero taxa. When attempting to minimize a taxon, minus the taxon frequency was used in the loss.

The 30 donors with the highest loss in the maximization task were chosen for the next generation creation (Fig. 6A, step D). To complete the donors’ parents of the next generation a mutation (see the “Methods” section) occurred with a probability of 0.3, and recombination (see “Methods” sections) occurred with a probability of 0.3 (Fig. 6A, step E) until a stopping criterion was achieved.

Even in the mouse model, where the difference between donor and recipient is very large, the GA converges within 25 generations in the optimization tasks (with $\gamma = 0$) of the Shannon diversity (Fig. 6B, C). We thus ran the GA for 25 generations for all the tasks. The results of the Shannon for different numbers of non-zero taxa are shown in Fig. 6D. The GA achieves a high Shannon with $\sim$ 100 different non-zero taxa (maximum $= 5.5,$ average $= 5$). The maximum was similar to the maximum of the existing data $(= 5.48)$ and the average was in the highest percentile of the distribution.

Succeeding to optimize the outcome while limiting the number of non-zero taxa opens the way for artificial transplant using a limited number of taxa. To check that the GA did not converge into trivial solutions, such as just generating donors with the targeted outcome, we calculated the SCC between the predicted targeted property and the property of the optimized donors generated. The SCCs were quite low, $|SCC| < 0.2$ (Fig. 6E).

We tested whether specific orders were consistently dominant in the optimized donors both in the Shannon diversity maximization tasks and in the orders’ relative abundance maximization tasks. In the Shannon maximization tasks, most of the orders varied; however, several orders were consistently frequent in the predicted transplant, especially for high $\gamma$ values, such as Bacteroidales, Clostridiales, and Fibrobacteriales (Fig. 6F). In the orders’ maximization tasks, the orders used by the GA are consistent among the different order tasks, such as Bifidobacterials, Verrucomicrobiales, and Methanobacteriales. The input orders were not directly related to the order that is maximized (Fig. 6G). For example, Lactobacillales affected Clostridiales, Enterobacterialles and its own frequency in the post-FMT recipient, and Clostridiales affected Bacillales, Bacteroidales, Desulfovibrionales, Verrucomicrobiales, Burkholderiales, Turicibacterales and Enterobacterialles.

## Discussion

FMTs are currently being tested in clinical trials as an emerging treatment for a wide range of disorders, including Parkinson’s disease, fibromyalgia, chronic fatigue syndrome, myoclonus dystopia, multiple sclerosis, obesity, insulin resistance, metabolic syndrome, and autism [65–72]. There are many open questions in FMT, including donor selection and screening, standardized protocols, long-term safety, and regulatory issues. The best method of treatment is also still being studied as some studies include antibiotic pre-treatment or bowel flushing prior to FMT while others forgo any pre-treatments, and the ramifications are still not fully understood [73–75]. Donor selection criteria also involve a number of practical and ethical considerations [76–78]. Non-autologous FMTs carry the possibility of transmitting infectious agents, and, therefore, rigorous screening tests are recommended to reduce infection risks. Such screenings limit the dangers of FMT but do not optimize their outcomes.

When optimizing for an outcome, the donor’s microbiome, physical activity, diet, drug use, medications, genetic background, age, sex, and a plethora of other factors all affect microbiota composition. Thus, it may be beneficial to consider the health profile of the donor. However, even with the ideal donor, the FMT success is not guaranteed [55, 79]. Another donor-related criterion that is considered is the similarity of microbial species expected between the recipient and the donor so that the mucosal adaptive immune system of the recipient presents more tolerance towards the microbiota from the donor [68, 80].

An alternative approach would be the generation of off-the-shelf donors expected to optimize the FMT engraftment probability and the expected improvement in clinical symptoms. However, there is currently no model for donor-microbiome-based selection of optimal donors.

To address this, we developed a tool to predict recipients’ post-FMT properties in human and mouse recipients using only the donor properties (microbiome composition, and demographics) and validated the prediction accuracy in a de-novo FMT experiment. The outcome predicted was either properties of the recipient’s post-FMT microbiome or improvement in clinical symptoms (for example response to IBD treatment as measured by the Mayo score). We further built a planned transplant of specific taxa while balancing between the number of non-zero taxa (cost and feasibility) and the quality of the optimization by using a GA.

To our knowledge, these are the first tools to propose a generic fully donor-based prediction for FMT success. Such algorithms can change the way FMT donor selection is performed―from randomly matching the donor or using a rational donor selection [81] to optimize the most appropriate donor given a certain outcome. The tools are available at:https://github.com/oshritshtossel/iMic_FMT and the trained algorithm (iMic) is available in the Drive at https://drive.google.com/file/d/1FIDy8uUBdv9Alj-xTe9Brkl5_QGBwamc/view?usp=sharing as well as in the Supplementary Material―“shannon weight.ckpt” for convenient reference and utilization.

The proposed models focused on the short-term post-FMT outcome. Longitudinal analyses in patients who have received FMT for recurrent CDI have shown an effect of FMT-induced microbiota alterations lasting anywhere from a few days to a few years after transfer [40, 82, 83]. A recent FMT/CDI study by Moss et al. discovered that despite the short-term similarity between donor and recipient gut microbiota profiles, concordance was significantly reduced after a year [84]. In the FMT study by Moayeddi et al., 8 of the 9 ulcerative colitis patients who were in remission at week 7 post-FMT were still in remission a year later with no instances of relapse [85]. In our results, the waning effect of the transplantation is accompanied by a decrease in the accuracy of the prediction, mainly in mice, and partially in humans.

While off-the-shelf treatments require a fully donor-based transplant selection, the recipient microbiome and health have been shown to also affect the outcome. Here, we observed a clear hierarchy in the outcome prediction accuracy, where GF mice are more precisely predicted than ABX-treated mice and ABX-treated humans are more precisely predicted than non-treated humans.

The danger of microbial toxicity may be solved via bacterial cocktails and personalized probiotics, in which we generate the transplant from scratch [17, 86, 87]. However, to our knowledge, generating a high variety of taxa from scratch has not yet been done commercially. We propose here an optimal solution for generating, such a mixture. Note that in all the models tested here, the donor properties were more important than other features, and there was a limited contribution of the recipient microbiome to the outcome (Fig. 5H). However, this may be a limitation of the datasets studied here.

To summarize, we have here proposed 2 alternative clinical scenarios. The first case is the choice among a set of existing donors for the specific donors optimizing the transplant outcome. The second is the possibility of generating a microbial “Soup” with a limited number of microbes. While the second solution may be safer and more efficient, the first one is probably more amenable to clinical use with current practices. The trained algorithm (iMic) is available in the Drive at https://drive.google.com/file/d/1FIDy8uUBdv9Alj-xTe9Brkl5_QGBwamc/view?usp=sharing and as a Supplementary Material―“shannon weights.ckpt”.

## Conclusions

The donor’s phenotype differs from the recipient’s phenotype. However, the recipient’s future properties a week post-FMT in GF mice and for a period of up to 24 weeks post-FMT in humans can be predicted from the donor’s microbiome solely by using our prediction tool. We further proposed another tool to optimize the optimal transplant (bacterial cocktails). By using our predictor and a GA, one can control the balance between the number of taxa to transplant and the targeted outcome. We validated our predictor using a de-novo FMT experiment highlighting the possibility to choose transplants that optimize the required goals. Our tools may change the current FMT protocols both on transplants from existing donors as well as planned transplants (from scratch).

### Supplementary information

**Additional file 1.**

**Additional file 2.**

## Data Availability

All the data was previously published apart from the Allergy data and the FMT validation experiments that are not published yet, but can be found at https://github.com/oshritshtossel/iMic_FMT. All the code is available at https://github.com/oshritshtossel/iMic_FMT.
