# Supplementary material for: Recipient-independent, high-accuracy FMT-response prediction and optimization in mice and humans
Source: Microbiome. 2023 Aug 14;11:181. doi: 10.1186/s40168-023-01623-w (PMC10424414; doi:10.1186/s40168-023-01623-w)
Supplement: Supplementary file 2 — Additional file 1. [file 40168_2023_1623_MOESM1_ESM.pdf]

Supplementary Material and Methods

June 6, 2023

1 Methods

1.1 Experimental datasets

1.1.1 Datasets: FMT from humans to GF (germ-free) mice (human-to-GF)

To parameterize our initial model to identify an ideal FMT (fecal microbiota transplantation) donor profile without recipient data, we collected microbiome data (16S rRNA sequences) of both human donors and murine recipients from 4 human-to-GF mouse FMT experiments, referred to as human-to-GF cohorts and colored in light blue in all figures. The data used included: (1) ASVs of the intervention (stool transplant) and (2) ASVs of the recipient (mouse) at different time points post-FMT. Fecal samples were collected from the mice every week after the transplantation. The samples were sequenced at the Azrieli Faculty of Medicine, Bar-Ilan University as previously described [1]. The datasets included: (a) "gestational diabetes mellitus" or "GDM": stools from pregnant women with gestational diabetes and pregnant controls [1]; (b) "Baby": stools from infants that were and were not treated with antibiotics in the first days of life [2]; (c) "Allergy": stools from humans with IgE-mediated food allergies and healthy controls (data not published); and (d) "Chemotherapy": stools from women who had had breast or gynecological cancer and were undergoing adjuvant chemotherapy [3]. The datasets used are described in Table 1.

1.1.2 Datasets: FMT from humans to humans

We collected microbiome data (16S rRNA sequences) of both human donors and human recipients from 8 human-to-human FMT experiments, referred to as human-to-human cohorts and colored in light orange in the figures. The datasets used are described in Table 2. For the clinical outcome predictions (success is response vs failure of FMT treatment at different time points after the FMT). We used 5 different cohorts: (a) inflammatory bowel disease (IBD) [4], as measured by Mayo score 3 months after FMT [5], (b) irritable bowel syndrome (IBS) measured by a decline in symptoms, (c)the response to PD-1 therapy in patients with melanoma [6], (d) ulcerative colitis (UC) measured by simple clinical colitis activity index scores (where ≥ 2 is ill) [7], and (e) antibiotics resistance (AR) measured by a decline in symptoms [7]. For more details see Table 3.

1.1.3 Microbiome characterization (human-to-GF and human-to-ABX mice)

DNA was extracted from all human and mouse fecal samples, using the MagMAX Microbiome Ultra-Kit (Thermo Fisher, Waltham, MA) according to the manufacturer's instructions and following a 2-minute bead beating step (BioSpec, Bartlesville, USA). The V4 region of the bacterial 16S rRNA gene was amplified by polymerase chain reaction (PCR) using the 515F (AATGATACGGCGACCACCGAGATCTACACGCT) barcoded and 806R (TATGGTAATTGTGTGYCAGCMGCCGCGGTAA) primers [8] with a final concentration of 0.04% of each primer

and 0.5% of PrimeSTAR Max DNA Polymerase (Takara-Clontech, Shiga, Japan) in 50μl total volume. PCR reactions were carried out by 30-35 cycles of denaturation (95°C), annealing (55°C) and extension (72°C), with final elongation at 72°C. PCR products were purified using XP magnetic beads (Beckman Coulter, Indianapolis, IN) and quantified using the Picogreen dsDNA quantitation kit (Invitrogen, Carlsbad, CA). Samples were then pooled in equal amounts, loaded on 2% agarose E-Gel (Thermo Fisher, Waltham, MA), purified and sent for sequencing using the Illumina MiSeq platform (Genomic Center, Azrieli Faculty of Medicine, Bar-Ilan University, Israel).

1.1.4 Dataset download and processing

Most datasets studied here are publicly available in the NCBI website. Those were downloaded using a newly developed package YaMaS `github.com/YarinBekor/YaMAS`. The Yamas package is a comprehensive tool that combines various packages and tools, such as the SRA-toolkit `https://trace.ncbi.nlm.nih.gov/Traces/sra/sra.cgi?view=software`, QIIME2 [9], and DADA2 plugin [10]. Its primary function is to enable easy downloading of DNA (Deoxyribonucleic acid) datasets from the NCBI (National Center for Biotechnology Information) and ENA (European Nucleotide Archive). The package is designed to be user-friendly, efficient and simple, even for non-programmers. The package follows a straightforward process. First, it downloads the raw DNA dataset. It then selects the appropriate pipeline for data processing. To transform short reads into fastq files, the package utilizes the SRA toolkit. To process 16S data type, it uses DADA2 from the QIIME package for denoising and utilizes QIIME for clustering the data and creating taxonomy, OTU, and phylogeny tree files. Conversely, for shotgun data, the package relies on the metaphlan package [11] to cluster the data and generate all relevant files.

1.1.5 Data merging, preprocessing, and normalization

When combining datasets, we padded with zeros the ASVs that were missing in some of the datasets. We applied the MIPMLP preprocessing [12]. For the models' inputs, we merged the donors' ASVs to the species taxonomy, by the mean method and a log normalization was followed. Notice that we analyzed the human-to-GF and human-to-human datasets separately.

1.1.6 Model parameters: Identifying ideal FMT donors and predicting FMT outcomes

The first model aimed to predict the donor maximizing the post-FMT Shannon's diversity index. In addition, models were built to predict FMT outcome based on donor data alone, focusing on predictions of relative abundances of all orders, the relative abundance of the 50 or 100 most prevalent species in the recipient mouse or human samples, respectively, following FMT, and presence/absence of the 50 or 100 most prevalent species in the recipient mouse or human sample respectively following FMT. Specific considerations and methods are described below.

Shannon's diversity
As a measure of FMT donor quality, we used the Shannon alpha diversity (Shannon) of the mice samples (recipients) t days after the transplant, $s_{i,t}$. Shannon's diversity index quantifies the uncertainty in predicting the species identity of an individual that is taken at random from the dataset [13]. Mathematically, it is defined by Shannon diversity $= -\sum_{i=1}^{s} p_i \cdot ln(p_i)$, where p is the proportion (n/N) of individuals of one particular species found (n) divided by the total number of individuals found (N) and s is the number of species. High alpha diversity is related to health, while low alpha diversity is related to different diseases.

Order-level relative abundances
We predicted the relative abundances of the 10 (mouse) or 30 (human) different orders present in the recipients after the transplant, s_i, based on donor sample profiles alone (a model per order). The orders' relative abundances were defined by merging the data at the order level in the MIPMLP pipeline via the sum merge function. A relative normalization was applied to each vector (see above).

Species-level relative abundances
Similarly, we predicted the relative abundances of the 50 most prevalent species of the recipients after the transplant in mice, s_i, and of the 100 most prevalent species in human samples (a model per specie). A relative normalization was applied to each vector (see above).

Species-level prevalence
Finally, we predicted the binary presence or absence of the 50 most prevalent species from the mouse samples and of the 100 most prevalent species from the human samples after the transplant, s_i (a model per specie).

FMT success
We also used our model to assess FMT outcomes in human-to-human FMT 5 clinical cohorts such as IBD measured by the improvement in Mayo score, response to PD-1 treatment in melanoma patients, etc.

1.2 Predictive models

When predicting outcomes, the time of the outcome was one of the input features of the model. For the training of the models, all the time steps of each sample i, t_i in the training set were used.

We also added metadata (donor's age, sex, and weight) when available. The missing metadata was completed by the median for the continuous variables, and by another category for the binary ones.

Linear predictors
We predicted the recipient outcomes at each time point after the FMT, s_{i,t_i} using some models implemented in sklearn, such as: a KNN (K nearest neighbors) regressor, SVR (Support Vector Machine regression) and Ridge regression [14, 15, 16]. The inputs of the models were the preprocessed ASVs from MIPMLP, a_i and a scalar of the number of days after the FMT t_i. We used a grid search over human to GF datasets and used the same hyperparameters for all the order and specie tasks. The same hyperparameters were used for all the tasks of the human-to-human datasets. No hyperparameter tuning was applied for the human-to-human tasks. For the best hyperparameters, see Supp. Mat. Table S1 and for the search space see Table S4.

Decision tree models
The inputs of the models were the preprocessed ASVs from MIPMLP, a_i and a scalar of the number of days after the FMT t_i. We used grid search in order to optimize the hyperparameters only on the Shannon task on the human to GF datasets and used the same hyperparameters for all the order and species tasks. The same hyperparameters were used for all the tasks of the human-to-human datasets. No hyperparameter tuning was applied in the human-to-human tasks. For the best hyperparameters, see Supp. Mat. Table S2 and for the search space see

Table S5. We used the XGBOOST regressor of *xgboost* [17] as well as the Random Forest (RF) regressor and classifier of *sklearn* [18].

Fully connected neural networks
We used two fully connected neural networks on the processed ASVs from the MIPMLP a_i and a scalar of the number of days after the FMT t_i. We used dropout L1 and L2 regularization to the network to avoid overfitting. We ran Neural Network Intelligence (NNI) [19] to find the best activation function and the number of neurons in each layer only on the Shannon task over the human-to-GF cohorts and used the same hyperparameters for all the order and specie tasks. The same hyperparameters were used for all the tasks of the human-to-human datasets. No hyperparameter tuning was applied for the human-to-human tasks. For the best hyperparameters, see Supp. Mat. Table S2 and for the search space see Table S5.

iMic - Image Microbiome
iMic translates the microbiome ASVs vectors to images, by using the structure of the taxonomy tree and the relations between the taxa in each group [20]. Once the donors' microbiome input was translated to an image, we used CNNs followed by two fully connected layers for the prediction itself. We fine-tuned the model's hyperparameters using an NNI framework on 10 cross-validations only on the Shannon task over the human-to-GF cohorts. We then used the same hyperparameters for all the order and specie tasks. The same hyperparameters were used for all the tasks of the human-to-human datasets. No hyperparameter tuning was applied for the human-to-human tasks. The best hyperparameters used are in Supp. Mat. Table S3 and the search space can be found in Table S6. We further used iMic with the same hyperparameters with additional metadata of the donors (concatenated to the flattened CNNs' output before the fully connected layers), including the donors' age, sex and weight at the transplant. We used the same metadata to predict all the time points after the FMT. We filled the missing data of the continuous variables by the variable median of the cohort. There was no missing data in the categorical variables. Notice that for the success-failure predictions, we used the same hyperparameters of all the human-to-human tasks. No further hyperparameter tuning was applied.

1.3 Experimental setup

1.3.1 Training test split

The data was divided into three groups. The training set consisted of 60% of the data, a validation set that contained 20% of the data for hyperparameter tuning (in the human to GF cohort only) and early stopping and an external test set of another 20% of the data, for the task evaluation (on which all the results are reported here). During the split, we ensured all the samples of the same mouse were in the same training, validation, or test set to prevent data leakage.

For all learning tasks, we used 10 cross-validations for the stability of the results. The results were reported as an average of 10 runs on the external test set.

1.3.2 Hyperparameter tuning

We computed the best hyperparameters for each model (KNN, SVR, LR, RF, XGBOOST, NN and iMic models) over the human-to-GF cohorts on the Shannon diversity task using a 10-fold cross-validation [21] on the internal validation only for the Shannon prediction. The same hyperparameters were used for all the other taxa frequency prediction tasks. We chose the hyperparameters according to the average R2 on the 10 validations. The platform we used for the optimization of the hyperparameters was NNI for the more complex models (NN, XGBOOST,

RF, and iMic models). For the simple models (KNN, SVR and Ridge), a grid search was applied. The tuned hyperparameters were: the coefficient of the L1 loss, the weight decay (L2-regularization), the activation function (RelU,elU or tanh, which makes the model non-linear), the number of neurons in the fully connected layers, dropout (a regularization method which zeros the neurons in the layers in the dropout's probability), batch size and learning rate. For the CNN models, we also had the kernel sizes as well as the strides and the padding as hyperparameters. The search space we used for each hyperparameter were: the L1 coefficient was chosen uniformly from [0,1]. The weight decay was chosen uniformly from [0,0.5]. The learning rate was one of [0.001,0.01,0.05]. The batch size was [32, 64, 218, 256]. The dropout was chosen universally from [0,0.05,0.1,0.2,0.3,0.4,0.5]. We chose the best activation function from RelU, ElU and tanh. The number of neurons was chosen relative to the input dimension. The first linear division factor from the input size was chosen randomly from [1,11]. The second layer division factor was chosen from [1,6]. The kernel sizes were defined by two different hyperparameters, a parameter for its length and its width. The length was in the range of [1,8] and the width was in the range of [1,20]. The strides were in the range of [1,9] and the channels were in the range of [1,16]. For the search space tables see Supp. Mat. Tables S4-S6. No hyperparameter tuning was done for the human-to-human tasks. We used the appropriate hyperparameters from the human-to-GF tasks.

1.4 Validation experiment

1.4.1 Identifying most and least optimal donors for the FMT validation experiment

Three groups were defined from the predicted properties (plotted as two groups for the sake of simplicity in Fig. 3 A). The first group is samples predicted to have a high Shannon after the FMT and the second group is predicted to have a low one (representing the top and bottom 20 % of the expected values. Given the association between the donor age and the transplant outcome, and that the entire second group was comprised of child donor samples, we also identified a third group, age-matched to the first, but expected to result in low diversity in donors (though not as low as the second group).

1.4.2 FMT experiment

After mice were pretreated with antibiotics and randomized into groups, FMT was administered via oral gavage as previously described [22]. Briefly, several grams of human donor fecal matter were resuspended in sterile PBS. 200μl of the solution was then delivered directly to the mouse's stomach using a flexible feeding tube. Two mice received FMT from each donor and then were housed together. The FMT was repeated for a second and final time 1 week after the first FMT to improve engraftment. Fecal samples were collected for microbiota profiling, and mice weights were recorded before each FMT and once per week for the six weeks following the second FMT, after which the mice were euthanized. Fecal microbiota changes were profiled through the experiment following FMT, and data were used to test model predictions of (1) Shannon diversity and also of another focal parameter - relative abundances at the (2) order level.

1.5 Synthetic community compilation

1.5.1 Genetic Algorithm (GA)

100 simulated "parent" donor populations were randomly chosen from all the simulated donor populations of all the cohorts (human-to-GF), a_i (Fig. 6 A. The following steps were then applied: step A). A binary representation, b_i, of the MIPMLP preprocessed donor vectors, a_i, was created

for each donor (Fig.6 A step B). Each MIPMLP preprocessed donor vector, a_i, got into the pre-trained iMic model with a scalar of 7 days after FMT. iMic returned the predicted outcome of each recipient (Fig. 6 A, step C). All the predicted outcomes, s_i, were used for the fitness function for selection. We used the following fitness function:

$$fitness_{max}(s_i, b_i) = s_i - sum(b_i) \cdot \gamma, \tag{1}$$

such that $sum(b_i)$ represents the number of non-zero taxa in the donor sample, and γ is a hyperparameter that controls the importance of solving the problem with minimum non-zero taxa. For the minimization tasks, we used the following fitness function:

$$fitness_{min}(s_i, b_i) = s_i + sum(b_i) \cdot \gamma, \tag{2}$$

such that $textsum(b_i)$ represents the number of non-zero taxa in the donor sample, and γ is a hyperparameter that controls the importance of solving the problem with maximum non-zero taxa.

Note that we do not explicitly require a given number of taxa in the optimized microbe combination. Instead, we penalize that by assuming a cost to each different microbe. As such, a new microbe is only added if it significantly improves the required outcome. Also, the limitation is distinct for each candidate donor. Each taxon can be separately present in each candidate combination.

To complete the donors' parents of the next generation a **mutation** (see below) occurs with a probability of 0.3, and a **recombination** (see below) occurs with a probability of 0.3 (Fig. 6 A, step E). Then the stopping rule is checked. If the stopping rule is met, the donors of step E are returned; otherwise, the new generation of donors from step E is the initial values in step C, till the stopping rule is met. The GA was applied for 25 generations since it converged within this number of generations. In mutations, a zero entry of the binary vector b_i from the 30 new optimized donors is replaced by one vector with a probability of 0.3. Then the entry of the MIMPLP preprocessed ASVs vector, a'_i is changed, respectively. In recombinations, half of a sample a_i and half of a sample a_j from the 30 new optimized donors are concatenated into a new recombined sample, $a*_k$.

2 FMT studies analysis

2.1 Microbiome properties distribution analysis

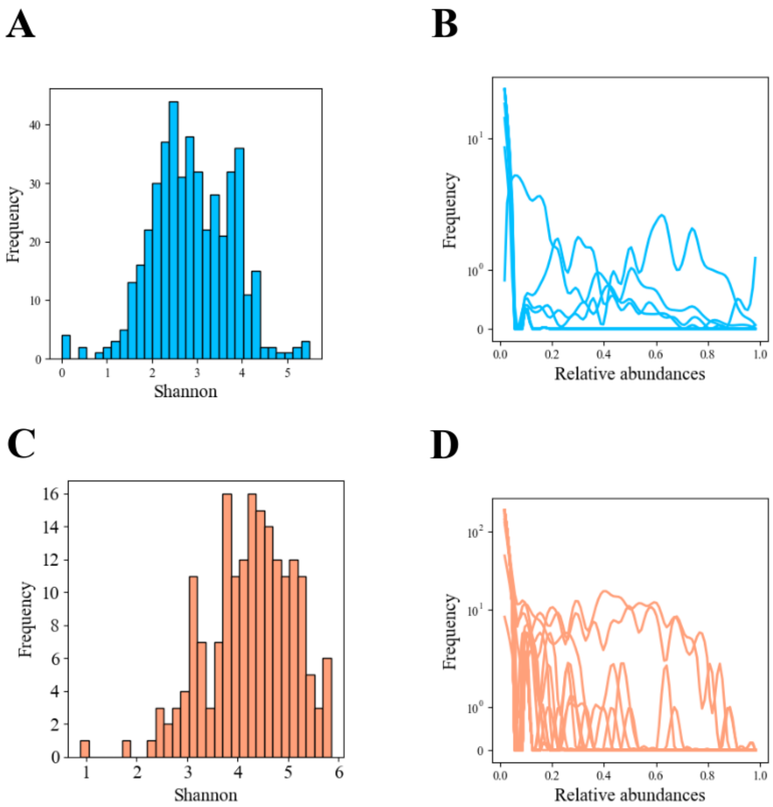

Figure 1: **A, C.** Shannon distributions in human-to-GF cohorts **(A)** and in human-to-human cohorts **(C)**. **B, D.** Overall orders distributions in human-to-GF cohorts **(B)** and human-to-human cohorts **(D)**.

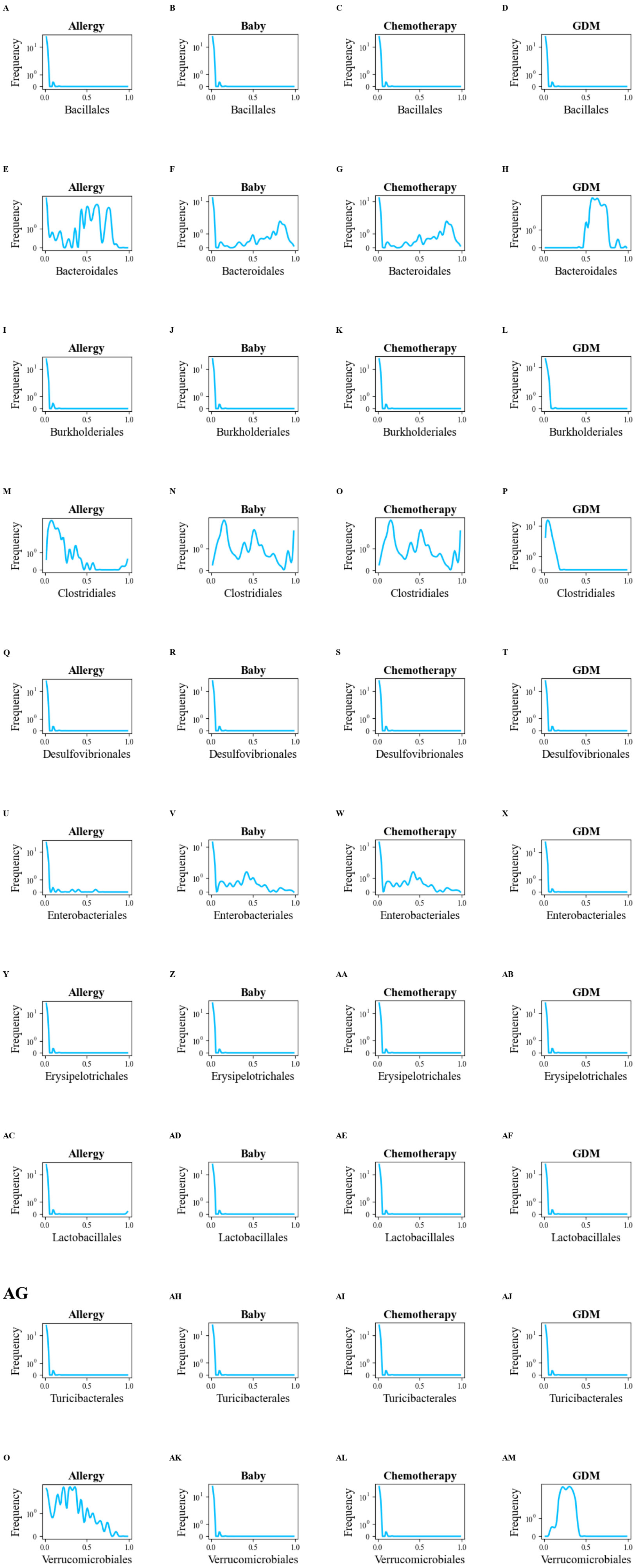

Figure 2: Orders distributions in the human-to-GF cohorts. Some orders distributions are consistent over the different cohorts, such as Bacilles and Desulfovibrionales, while others are not, such as Bacteroidels and Clostridiales.

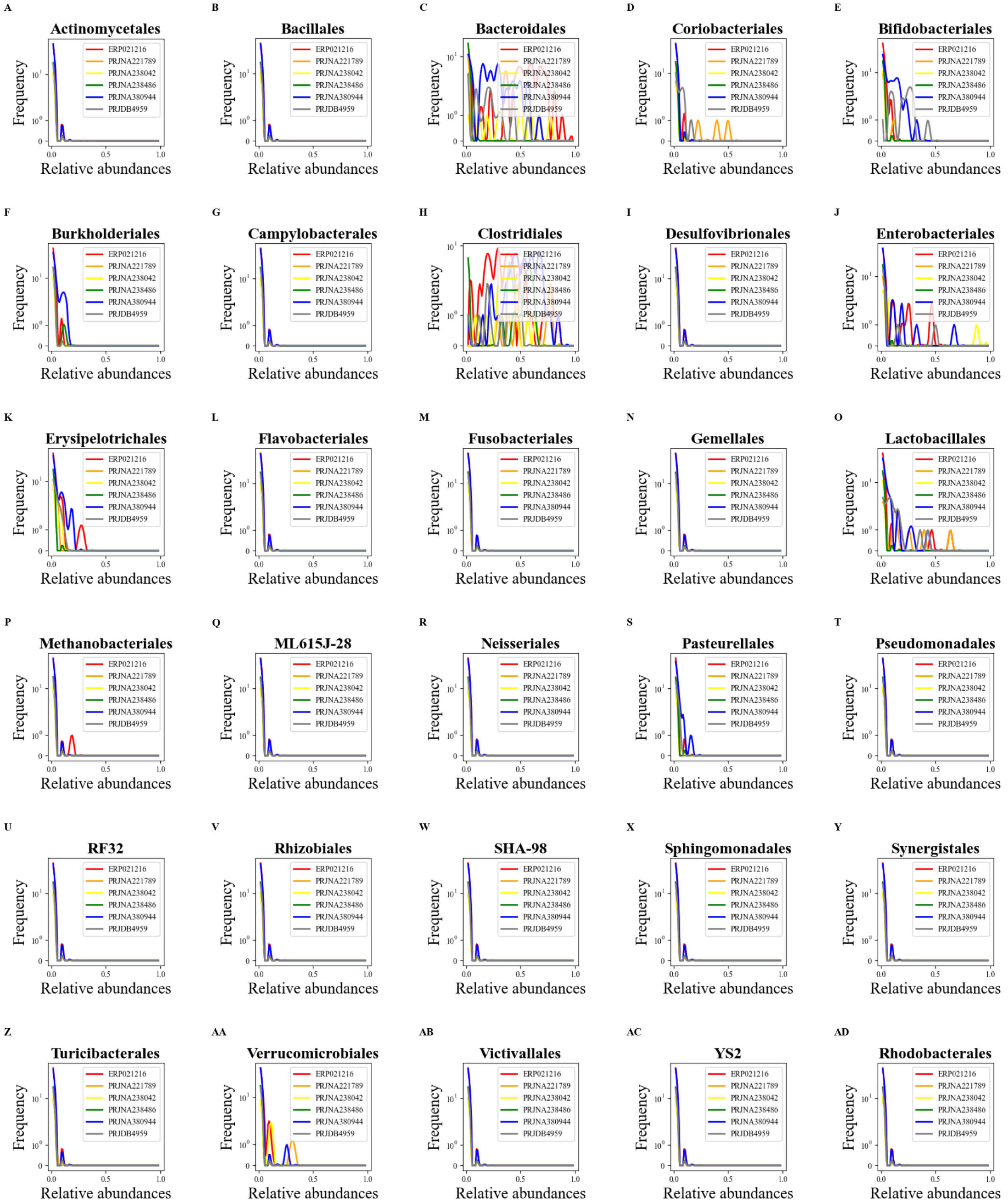

Figure 3: Orders distributions over the datasets of the human-to-human cohort, as in the human-to-GF cohorts.

2.2 Limited relation between donor and recipient properties.

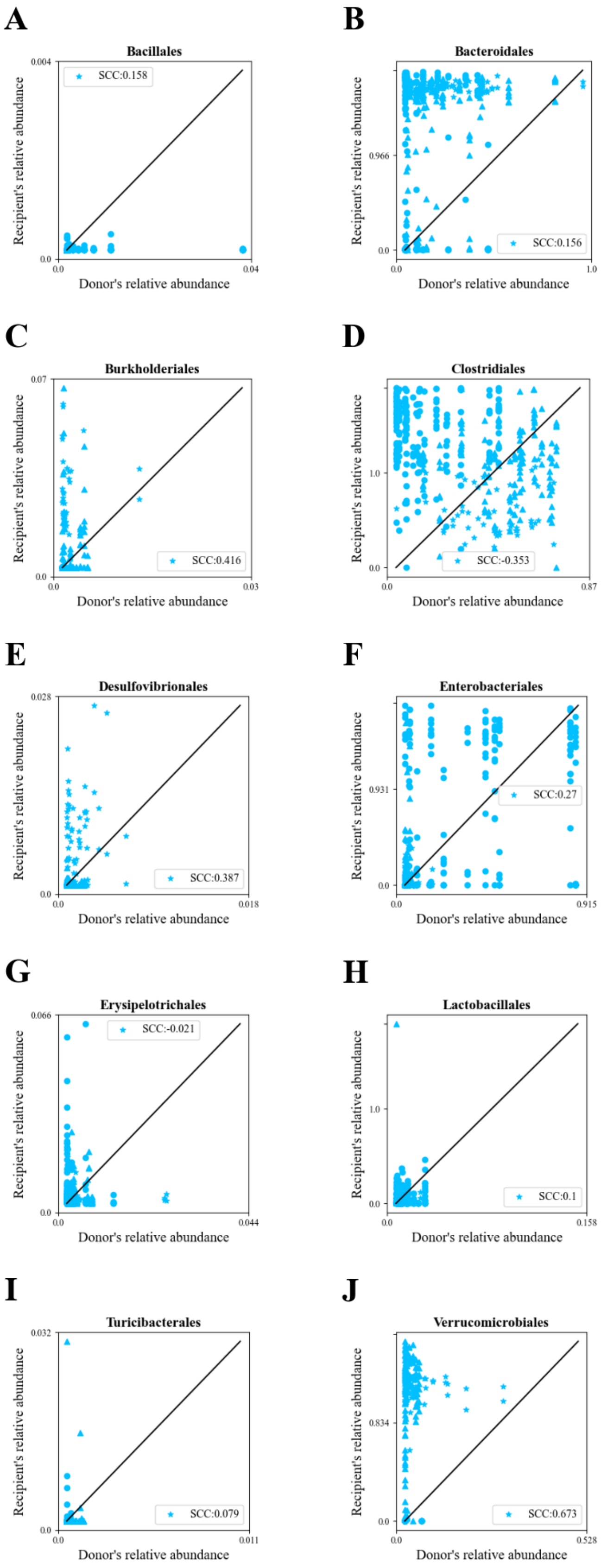

Figure 4: Limited relation between donor and recipient properties. **A-J.** Scatter plots of donor's property vs recipient's property. The black line represents equal values. Each shape represents a cohort. The dots represent the Baby dataset, the stars represent the GDM dataset, the Xs represent the Chemotherapy dataset, and the triangles represent the Allergy dataset.

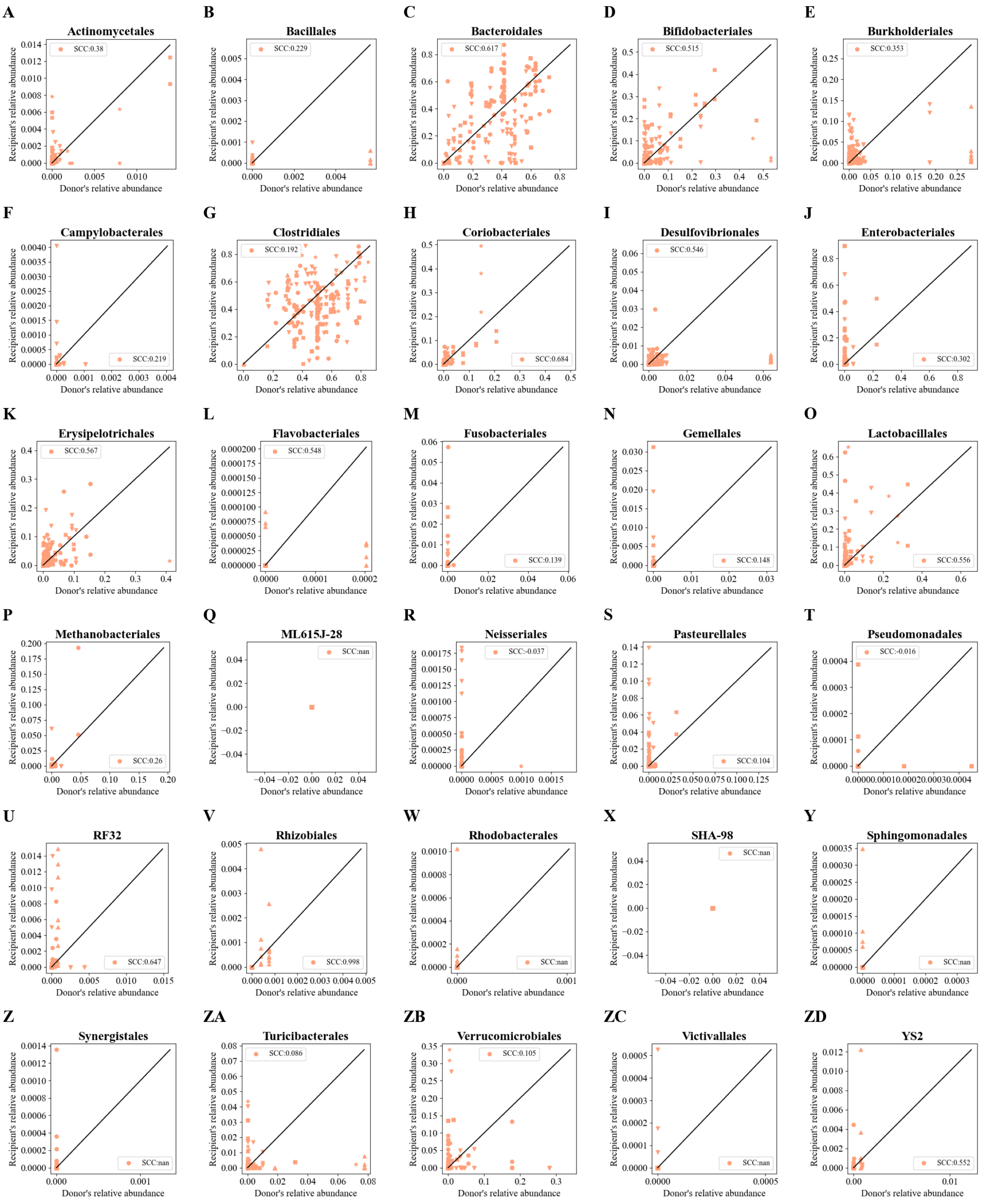

Figure 5: Limited relation between donor and recipient properties in the human-to-human analysis. **A-ZD.** Scatter plots of donor's property vs recipient's property. The plot is similar to the plot above. Orders with 0 values for all recipients or all donors were removed. Each shape represents samples from different cohorts as assigned in Fig. 1 C.

3 The recipient's properties can be predicted only from the donor's sample

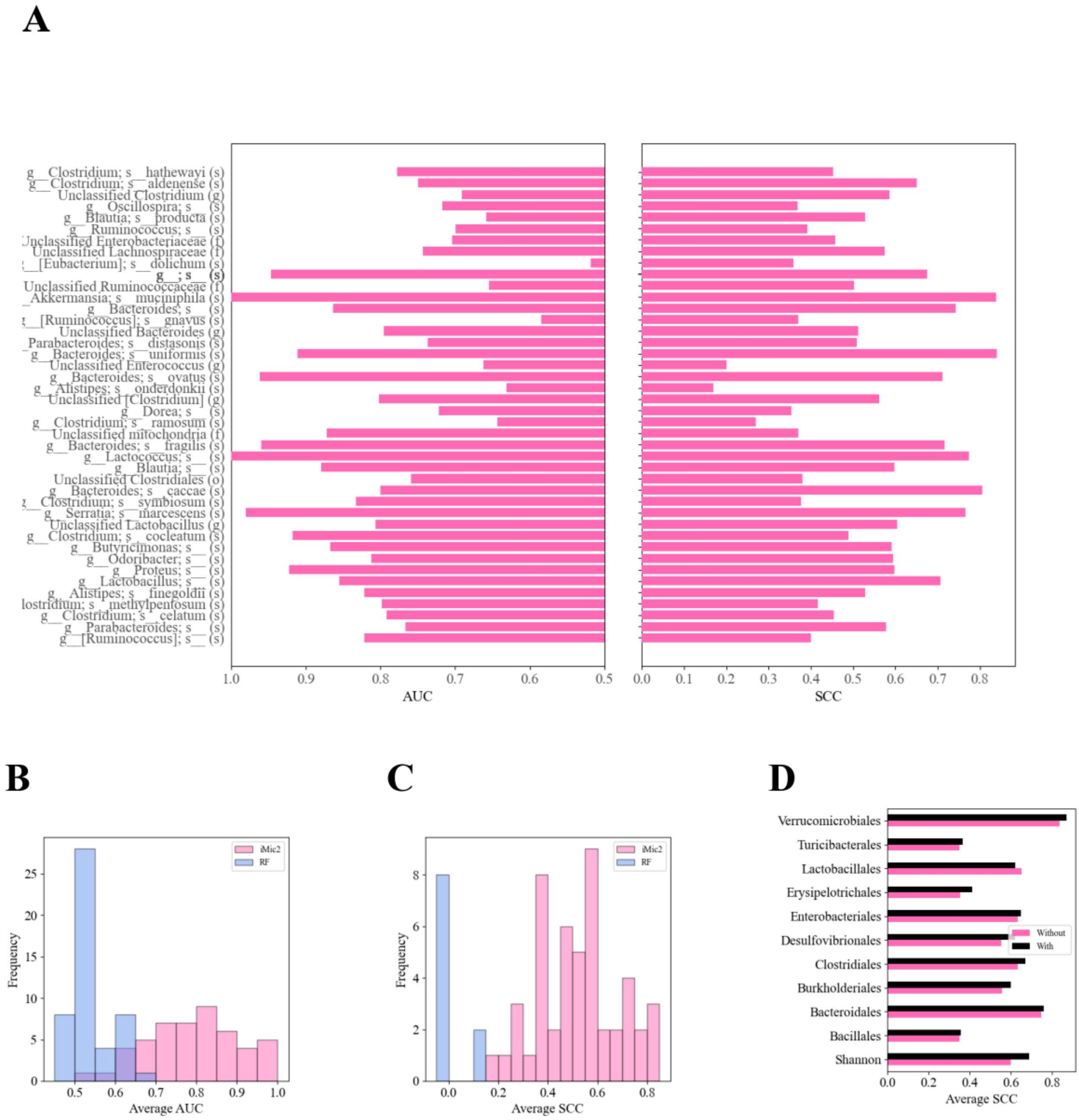

Figure 6: The recipient's properties can be predicted only from the donor's sample in human-to-GF cohorts. **A.** Average AUC (of presence-absence predictions) and SCC (Spearman correlation coefficient) (of relative abundance predictions) over 10 cross-validations (CVs) of the 50 most frequent species in the cohorts. The left bars represent the AUCs and the right bars represent the SCCs. **B - C.** Histograms of average AUCs **(B)** and average SCCs **(C)** of iMic2 and RF, where iMic2's results are in pink and RF's results are in light blue. In the relative abundances predictions (most of the species results are absent for the RF since it predicts 0 abundance for all the samples. **D.** Adding metadata of age, sex, and weight to the iMic2 model improves the prediction results of the Shannon and the order's relative abundances. The x-axis represents the model, the y-axis represents the average SCC over 10 CVs. The black bars represent the learning with the metadata, and the pink bars represent the original learning with no added metadata.

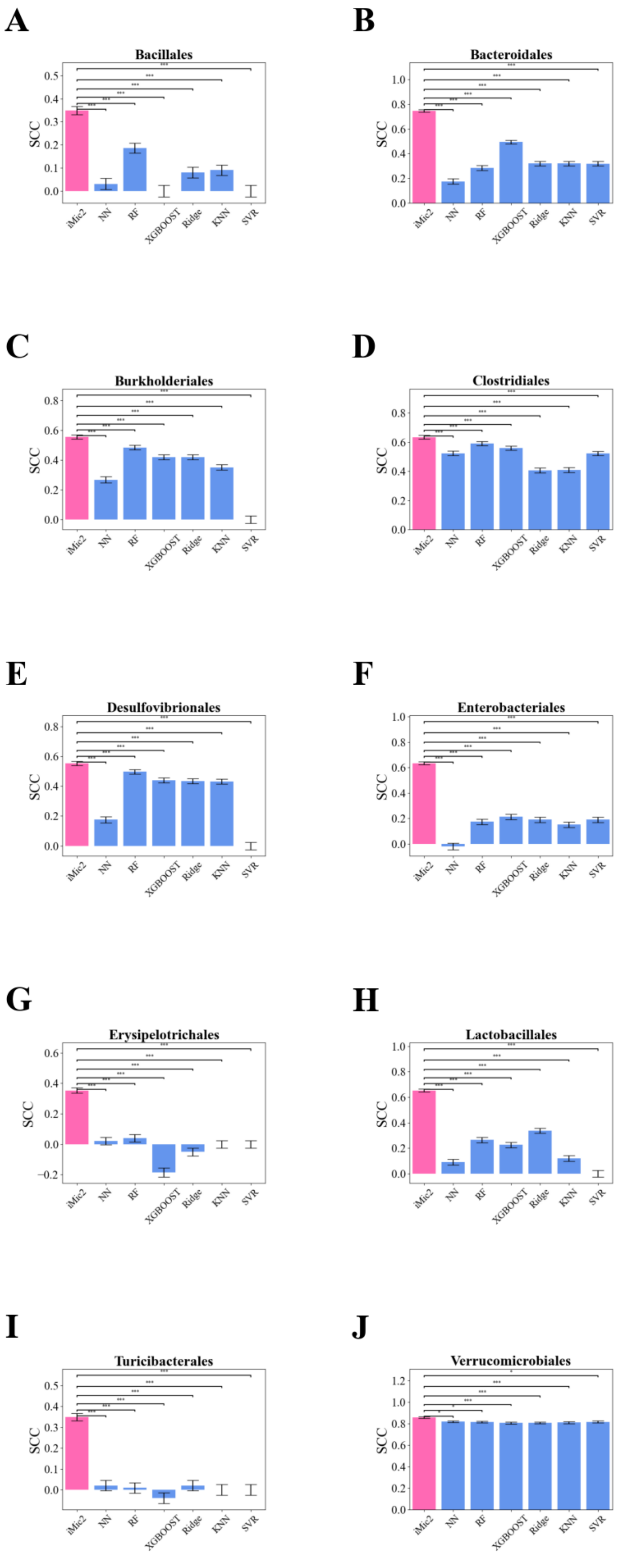
A
Bacillales
B
Bacteroidales
C
Burkholderiales
D
Clostridiales
E
Desulfovibrionales
F
Enterobacteriales
G
Erysipelotrichales
H
Lactobacillales
I
Turicibacterales
J
Verrucomicrobiales
SCC
iMic2
NN
RF
XGBOOST
Ridge
KNN
SVR

Figure 7: Recipient's orders can be predicted from donor's samples only (humans-to-GF). Different models' evaluation scores of recipient's relative abundances SCC. The x-axis represents the model. The Ridge, KNN and SVR models are in **blue**, the networks and trees - XGBOOST and NN are in **blue**, and the structure-based CNNs - iMic-2 is in **pink**. The standard errors (error bars) over the 10 CVs are in black. The stars represent the significance of p-value * - $p \leq 0.05$, ** - $p \leq 0.01$, *** - $p \leq 0.001$.

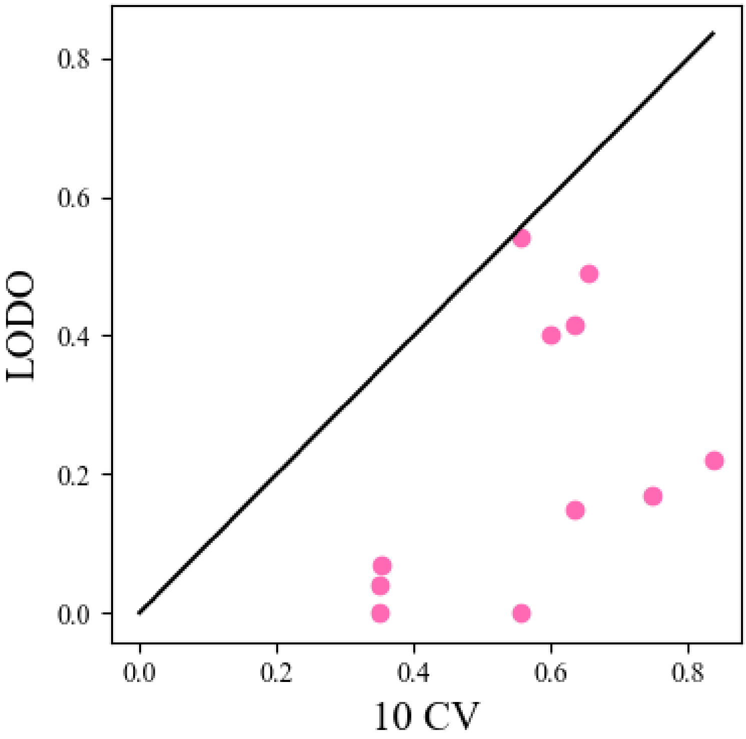

Figure 8: 10CV vs LODO (leave-one-dataset-out). The x-axis represents the average SCC over 10 CVs of the iMic model on a mixed dataset (human-to-GF). The y-axis represents the SCC of an iMic trained on 3 datasets (Allergy, GDM and Baby) and tested on the Chemotherapy dataset. The black line is $y = x$, where the SCC of the CVs is equal to the SCC of the LODO.

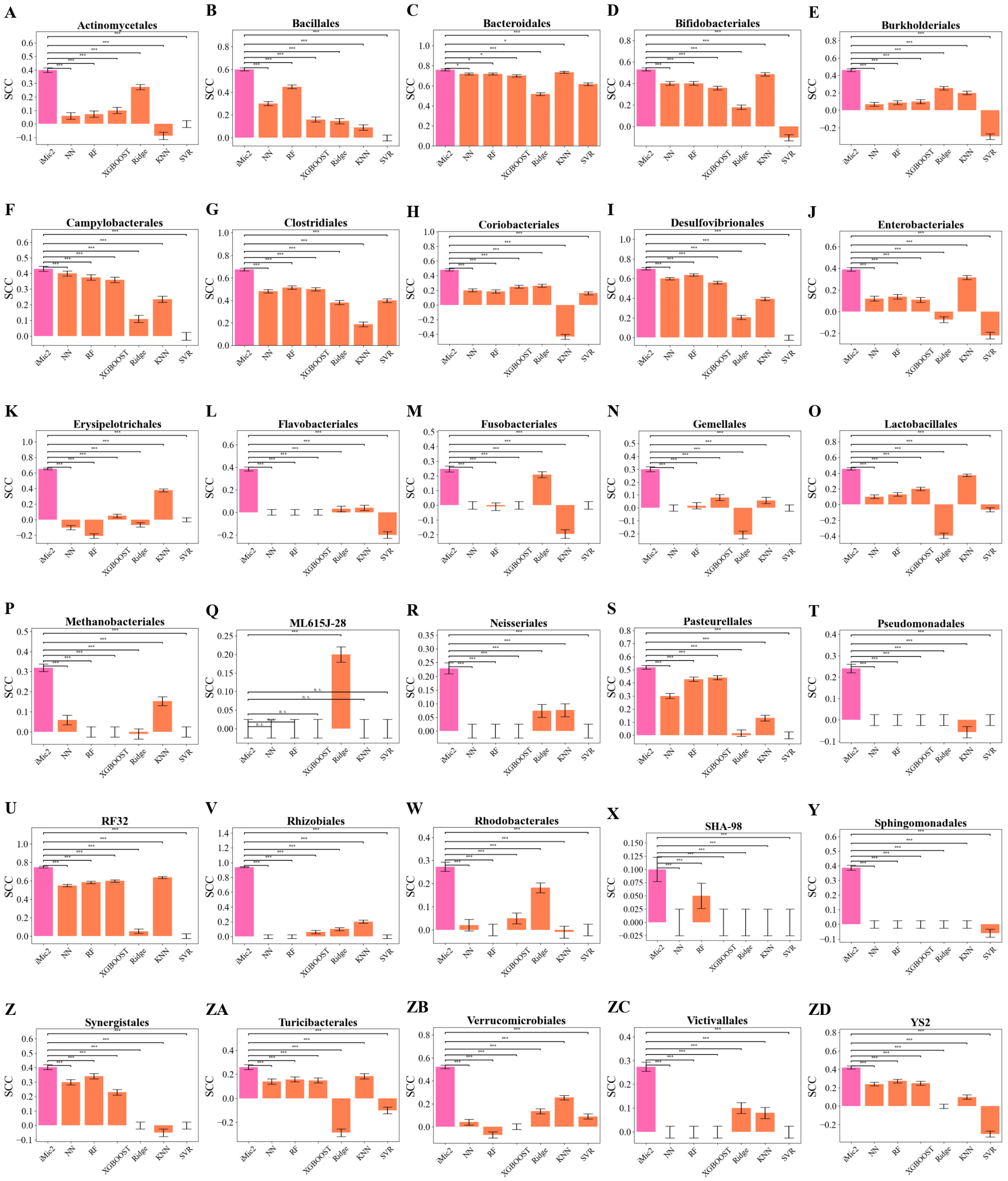

Figure 9: Recipient's orders can be predicted from donor's samples only (human-to-human datasets). Different models evaluation scores of recipient's relative abundances SCC. The colors and symbols are as above (orange for the non-iMic models and pink for the iMic model).

4 Models hyperparameters

All models' hyperparameters were optimized on a validation set of 20% of the data. The search was applied by a grid-search to the simple models (KNN, SVR and Ridge) and by an NNI to the more complicated models (NN, XGBOOST,iMic1 and iMic2).

4.1 Hyperparameters used

Table 1: Simple models' (KNN, SVR, Ridge) hyperparameters used.

	**KNN**	**SVR**	**Ridge**
**Number of neighbors**	5	-	-
**Distance metric**	minkowsky	-	-
**Regularization**	-	1	1
**Kernel**	-	rbf	-

Table 2: NN RF and XGBOOST's hyperparameters used

	**NN**	**RF**	**XGBOOST**
**Booster**	-	-	gbtree
Criterion	-	gini	-
**Number of trees**	-	-	100
**Learning rate**	0.001	-	0.3
**Max depth**	-	None	5
**L1 regularization**	0.59	-	0.5
**L2 regularization**	0.006	-	1.5
**Activation function**	-	-	elU
**Dropout**	-	-	0.05
**Linear dimension 1**	317	-	-
**Linear dimension 2**	126	-	-
**Batch size**	256	-	-

Table 3: iMic's models hyperparameters used.

	**iMic1**	**iMic2**
**Learning rate**	0.001	0.001
**L1 regularization**	0.632	0.563
**L2 regularization**	0.009	0.0005
**Activation function**	elU	relU
**Dropout**	0.3	0.1
**Linear dimension 1 (div)**	4	6
**Linear dimension 2 (div)**	5	11
**Batch size**	64	128
**Kernel size 1 first convolution layer (rows)**	3	4
**Kernel size 2 first convolution layer (columns)**	17	12
**Kernel size 1 second convolution layer (rows)**	-	2
**Kernel size 2 second convolution layer (columns)**	-	5
**Stride first convolution layer**	5	1
**Stride second convolution layer**	-	1
**Padding first convolution layer**	0	2
**Padding second convolution layer**	-	0
**Channels 1**	14	7
**Channels 2**	-	4

4.2 Search space fields of hyperparameters

Table 4: Simple models (KNN, SVR, Ridge) hyperparameters search space

	**KNN**	**SVR**	**Ridge**
**Number of neighbors**	{3,5,7,9}	-	-
**Distance metric**	{minkowski,euclidean,manhattan}	-	-
**Regularization**	-	[0,1]	[0,1]
**Kernel**	-	{rbf,linear,sigmoid,poly}	-

Table 5: NN RF and XGBOOST's hyperparameters used

	**NN**	**RF**	**XGBOOST**
**Booster**	-	-	{"gbtree","gblinear","dart"}
**Criterion**	-	{"gini","entropy","log_loss"	-
**Number of trees**	-	-	{10,50,100,150,200}
**Learning rate**	0.001	-	[0,1]
**Max depth**	-	{None,3,4,5,10,20}	{3,4,5,6}
**L1 regularization**	[0,1]	-	[0,1]
**L2 regularization**	[0,0.05]	-	{0,0.5,1,1.5,2}
**Activation function**	{"relU","elU","Tanh"}	-	-
**Dropout**	[0,0.5]	-	-
**Linear dimension 1**	[1,320]	-	-
**Linear dimension 2**	[1,200]	-	-
**Batch size**	{32,64,128,256}	-	-

References

[1] Yishay Pinto, Sigal Frishman, Sondra Turjeman, Adi Eshel, Meital Nuriel-Ohayon, Oren Ziv, Shtossel Oshrit, William Walters, Julie Parsonnet, Catherine Ley, Elizabeth L Johnson, et al. First trimester gut microbiome induces inflammation-dependent gestational diabetes phenotype in mice. *medRxiv*, 2021.

[2] Atara Uzan-Yulzari, Olli Turta, Anna Belogolovski, Oren Ziv, Christina Kunz, Sarah Perschbacher, Hadar Neuman, Edoardo Pasolli, Aia Oz, Hila Ben-Amram, et al. Neonatal antibiotic exposure impairs child growth during the first six years of life by perturbing intestinal microbial colonization. *Nature Communications*, 12(1):1–12, 2021.

[3] Atara Uzan-Yulzari, Maya Morr, Hala Tareef-Nabwani, Oren Ziv, Dafna Magid-Neriya, Ran Armoni, Efrat Muller, Anca Leibovici, Elhanan Borenstein, Yoram Louzoun, et al. The intestinal microbiome, weight, and metabolic changes in women treated by adjuvant chemotherapy for breast and gynecological malignancies. *BMC Medicine*, 18(1):1–10, 2020.

[4] Ruiqiao He, Pan Li, Jinfeng Wang, Bota Cui, Faming Zhang, and Fangqing Zhao. The interplay of gut microbiota between donors and recipients determines the efficacy of fecal microbiota transplantation. *Gut Microbes*, 14(1):2100197, 2022.

Table 6: iMic models search space.

	iMic1	iMic2
**Learning rate**	{0.001,0.01,0.05}	{0.001,0.01,0.05}
**L1 loss coefficient**	[0.1,0.8]	[0.1,0.8]
**L2 regularization**	[0,0.002]	[0,0.002]
**Activation function**	{"relU","Tanh","elU"}	{"relU","Tanh","elU"}
**Dropout**	[0,0.5]	[0,0.5]
**Linear dimension 1 (div)**	[1,11]	[1,12]
**Linear dimension 2 (div)**	[1,6]	[1,12]
**Batch size**	{32,64,128,256}	{32,64,128,256}
**Kernel size 1 first convolution layer (rows)**	[1,8]	[2,5]
**Kernel size 2 first convolution layer (columns)**	[1,20]	[3,15]
**Kernel size 1 second convolution layer (rows)**	-	[1,3]
**Kernel size 2 second convolution layer (columns)**	-	[1,10]
**Stride first convolution layer**	[1,9]	[1,6]
**Stride second convolution layer**	-	[1,5]
**Padding first convolution layer**	[0,4]	[0,4]
**Padding second convolution layer**	-	[0,4]
**Channels 1**	[1,16]	[1,10]
**Channels 2**	-	[1,17]

[5] James D Lewis, Shaokun Chuai, Lisa Nessel, Gary R Lichtenstein, Faten N Aberra, and Jonas H Ellenberg. Use of the noninvasive components of the mayo score to assess clinical response in ulcerative colitis. *Inflammatory bowel diseases*, 14(12):1660–1666, 2008.

[6] Diwakar Davar, Amiran K Dzutsev, John A McCulloch, Richard R Rodrigues, Joe-Marc Chauvin, Robert M Morrison, Richelle N Deblasio, Carmine Menna, Quanquan Ding, Ornella Pagliano, et al. Fecal microbiota transplant overcomes resistance to anti–pd-1 therapy in melanoma patients. *Science*, 371(6529):595–602, 2021.

[7] Noortje G Rossen, Susana Fuentes, Mirjam J van der Spek, Jan G Tijssen, Jorn HA Hartman, Ann Duflou, Mark Löwenberg, Gijs R van den Brink, Elisabeth MH Mathus-Vliegen, Willem M de Vos, et al. Findings from a randomized controlled trial of fecal transplantation for patients with ulcerative colitis. *Gastroenterology*, 149(1):110–118, 2015.

[8] J Gregory Caporaso, Christian L Lauber, William A Walters, Donna Berg-Lyons, James Huntley, Noah Fierer, Sarah M Owens, Jason Betley, Louise Fraser, Markus Bauer, et al. Ultra-high-throughput microbial community analysis on the illumina hiseq and miseq platforms. *The ISME Journal*, 6(8):1621–1624, 2012.

[9] Evan Bolyen, Jai Ram Rideout, Matthew R Dillon, Nicholas A Bokulich, Christian C Abnet, Gabriel A Al-Ghalith, Harriet Alexander, Eric J Alm, Manimozhiyan Arumugam, Francesco Asnicar, et al. Reproducible, interactive, scalable and extensible microbiome data science using qiime 2. *Nature biotechnology*, 37(8):852–857, 2019.

[10] Benjamin J Callahan, Paul J McMurdie, Michael J Rosen, Andrew W Han, Amy Jo A Johnson, and Susan P Holmes. Dada2: High-resolution sample inference from illumina amplicon data. *Nature methods*, 13(7):581–583, 2016.

[11] Duy Tin Truong, Adrian Tett, Edoardo Pasolli, Curtis Huttenhower, and Nicola Segata. Microbial strain-level population structure and genetic diversity from metagenomes. *Genome research*, 27(4):626–638, 2017.

[12] Yoel Jasner, Anna Belogolovski, Meirav Ben-Itzhak, Omry Koren, and Yoram Louzoun. Microbiome preprocessing machine learning pipeline. *Frontiers in Immunology*, page 1954, 2021.

[13] Ashwani K Thukral. A review on measurement of alpha diversity in biology. *Agric. Res. J*, 54(1):1–10, 2017.

[14] John Platt et al. Probabilistic outputs for support vector machines and comparisons to regularized likelihood methods. *Advances in Large Margin Classifiers*, 10(3):61–74, 1999.

[15] Donald W Marquardt and Ronald D Snee. Ridge regression in practice. *The American Statistician*, 29(1):3–20, 1975.

[16] Gongde Guo, Hui Wang, David Bell, Yaxin Bi, and Kieran Greer. Knn model-based approach in classification. In *OTM Confederated International Conferences "On the Move to Meaningful Internet Systems"*, pages 986–996. Springer, 2003.

[17] Tianqi Chen, Tong He, Michael Benesty, Vadim Khotilovich, Yuan Tang, Hyunsu Cho, Kailong Chen, et al. Xgboost: extreme gradient boosting. *R package version 0.4-2*, 1(4):1–4, 2015.

[18] F. Pedregosa, G. Varoquaux, A. Gramfort, V. Michel, B. Thirion, O. Grisel, M. Blondel, P. Prettenhofer, R. Weiss, V. Dubourg, J. Vanderplas, A. Passos, D. Cournapeau, M. Brucher, M. Perrot, and E. Duchesnay. Scikit-learn: Machine learning in Python. *Journal of Machine Learning Research*, 12:2825–2830, 2011.

[19] Microsoft Research. Neural Network Intelligence, 1 2021.

[20] Shtossel Oshrit, Isakov Haim, Turjeman Sondra, Koren Omry, and Louzoun Yoram. Image and graph convolution networks improve microbiome-based machine learning accuracy. *arXiv preprint arXiv:2205.06525*, 2022.

[21] Tadayoshi Fushiki. Estimation of prediction error by using k-fold cross-validation. *Statistics and Computing*, 21(2):137–146, 2011.

[22] Henni Hiltunen, Hila Hanani, Raakel Luoto, Sondra Turjeman, Oren Ziv, Erika Isolauri, Seppo Salminen, Omry Koren, and Samuli Rautava. Preterm infant meconium microbiota transplant induces growth failure, inflammatory activation, and metabolic disturbances in germ-free mice. *Cell Reports Medicine*, 2(11):100447, 2021.
